# Joint inference and alignment of genome structures enables characterization of compartment-independent reorganization across cell types

**DOI:** 10.1186/s13072-019-0308-3

**Published:** 2019-10-08

**Authors:** Lila Rieber, Shaun Mahony

**Affiliations:** Department of Biochemistry and Molecular Biology and Center for Eukaryotic Gene Regulation, The Pennsylvania State University, University Park, PA 16802 USA

**Keywords:** Hi–C, Structural inference, Gene regulation

## Abstract

**Background:**

Comparisons of Hi–C data sets between cell types and conditions have revealed differences in topologically associated domains (TADs) and A/B compartmentalization, which are correlated with differences in gene regulation. However, previous comparisons have focused on known forms of 3D organization while potentially neglecting other functionally relevant differences. We aimed to create a method to quantify all locus-specific differences between two Hi–C data sets.

**Results:**

We developed MultiMDS to jointly infer and align 3D chromosomal structures from two Hi–C data sets, thereby enabling a new way to comprehensively quantify relocalization of genomic loci between cell types. We demonstrate this approach by comparing Hi–C data across a variety of cell types. We consistently find relocalization of loci with minimal difference in A/B compartment score. For example, we identify compartment-independent relocalizations between GM12878 and K562 cells that involve loci displaying enhancer-associated histone marks in one cell type and polycomb-associated histone marks in the other.

**Conclusions:**

MultiMDS is the first tool to identify all loci that relocalize between two Hi–C data sets. Our method can identify 3D localization differences that are correlated with cell-type-specific regulatory activities and which cannot be identified using other methods.

## Background

Chromosome conformation data have become available for diverse cell types, perturbations, and developmental stages. Comparing these data sets can highlight the relationship between three-dimensional chromosomal structure and biological function. To date, such comparisons have primarily examined known chromosomal structures, such as A/B compartments, topologically associated domains (TADs), and loops. Though TADs are largely conserved across cell types and species [1], extensive differences in compartmentalization [2, 3] and looping are detectable between data sets [4–6] and are correlated with differential gene expression. However, focusing on known types of differences means that other potentially functional differences remain unexplored. For example, relocalization within a compartment or TAD could be correlated with differences in gene regulation but might not be identified by current approaches for comparing Hi–C data sets.

Current methods for comparing chromosome conformation data sets (primarily from Hi–C experiments) can be classified as global, interaction-specific, or locus-specific. Some global methods calculate an overall similarity score for two Hi–C data sets, enabling clustering of experiments [7–10]. Global concordance in TAD boundaries can also be calculated [11]. However, these methods cannot discover specific differences between data sets. Interaction-specific methods identify genomic locus pairs that significantly differ in their interaction frequency, which may indicate gain or loss of a chromatin loop [12–16], but cannot determine how the interacting pair of loci have moved with respect to the rest of the genome or which locus within the pair drives the change. Locus-specific methods identify differences in organization, such as a difference in compartment score [2, 3] or insulation score [17, 18], that occur at a single locus, which is a single bin at the resolution of the Hi–C data. However, these methods are currently limited to measuring differences in known forms of chromatin organization, preventing the discovery of novel structures. There is currently no method for quantifying general locus-specific relocalizations between Hi–C data sets. Ideally, such a method would quantify the degree to which a given locus has changed position with respect to the rest of the genome, regardless of whether the relocalization was driven by differences in compartmentalization, TAD structure, looping, or a combination of several effects.

In a Hi–C data set, each locus is represented as a vector of interaction frequencies with every other locus of the genome or chromosome. Because typical metrics for vector comparison, such as Pearson correlation, are biased by Hi–C distance decay, comparison of data sets is challenging [10]. To mitigate issues associated with the high dimensionality of Hi–C data, we first aim to embed the data sets in a lower dimensional space. In this work, we choose to embed Hi–C data in three dimensions, representing the population average chromosome structure. While the physical interpretation of 3D chromosome structures is limited by population heterogeneity, we propose that comparing two 3D structures provides a convenient and intuitive assessment of the overall differences in chromatin organization across cell types or conditions. If the structures are comparable and correctly aligned, the degree to which a given locus has shifted position can be calculated as the Euclidean distance between the 3D coordinates of that locus in each of the structures. We have developed MultiMDS to simultaneously infer and align 3D structures from two Hi–C data sets. By applying our method to a number of mammalian and yeast Hi–C data sets, we identified examples of chromatin relocalization correlated with biological function, some of which confirm previous findings and some of which are potentially novel.

## Results

### MultiMDS: a principled approach for comparing genome structures

**Fig. 1:**
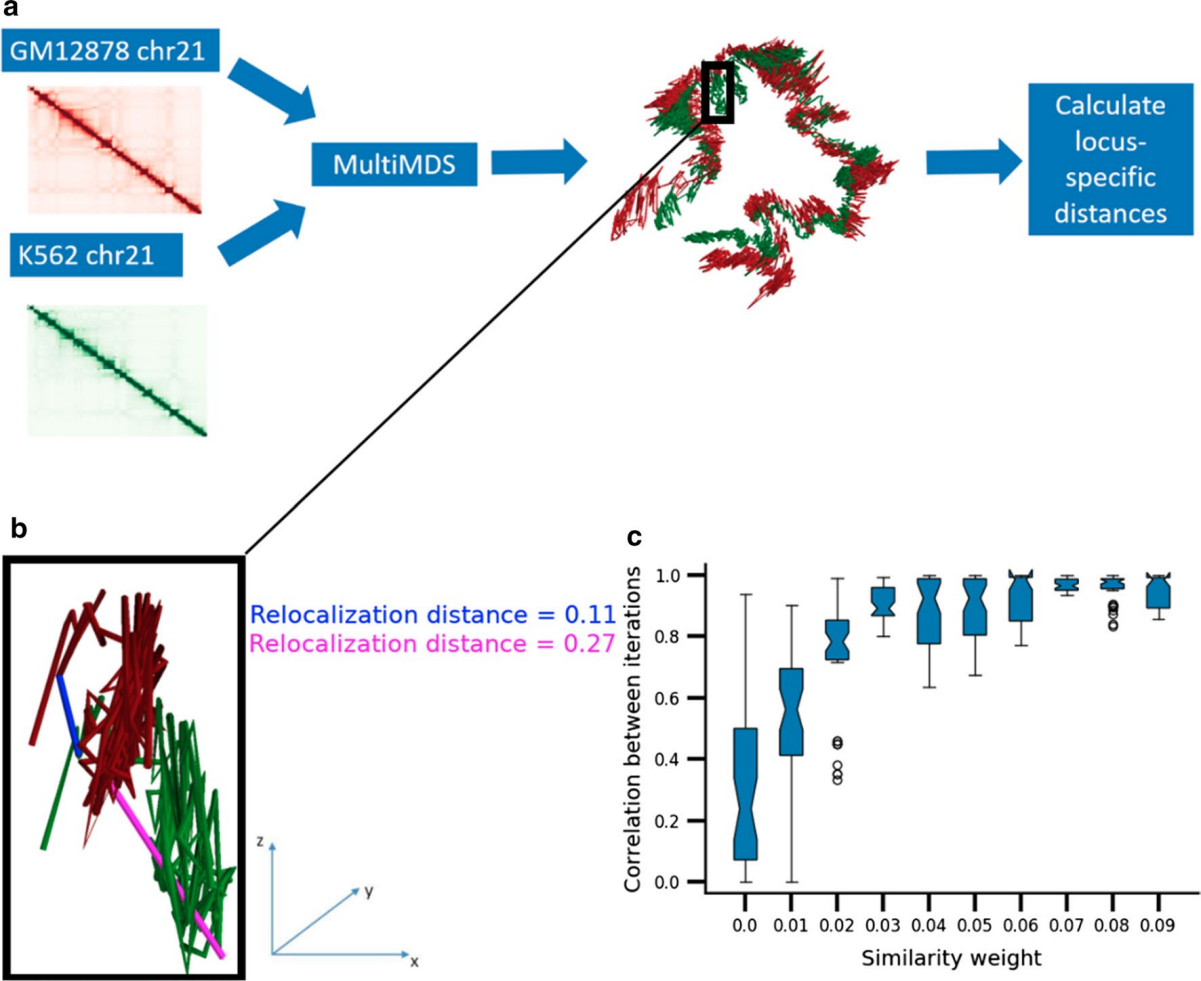
**a** Example of MultiMDS applied to GM12878 and K562 chr21 data sets at 10-kb resolution. **b** Example of relocalization distance calculations in aligned GM12878 and K562 chr21 structures. Only chr21:22.26–23.26 Mb is shown. Relocalization distance is calculated for the loci 22.61–22.62 Mb (magenta) and 22.31–22.32 Mb (blue). **c** Pairwise correlations between multiple runs of MultiMDS applied to GM12878 and K562 chr21, measured across a range of similarity weights. Zero weight represents independent inference and alignment

We developed MultiMDS to quantify locus-specific relocalization between Hi–C data sets. MultiMDS takes as input two normalized Hi–C contact matrices and outputs two aligned 3D structures, which represent the ensemble average structures for the respective inputs (Fig. 1a). Relocalization is calculated as the locus-specific Euclidean distance between aligned structures (Fig. 1b). Because Hi–C contact frequencies are believed to be a function of the average physical distances in the chromosomal structures, a distance-preserving dimensionality reduction method is the most intuitive option. Multidimensional scaling (MDS) minimizes the difference between the input distances derived from the Hi–C contact matrix and the embedded distances, so it has previously been used for structural inference from Hi–C data [19–23]. Though it is possible to use other dimensionality reduction methods, such as principal component analysis (PCA), to embed Hi–C data, these methods are often not distance-preserving and so the relationship between structural distances and physical distances is less clear.

It is possible to align independently estimated structures, for example [24]. However, due to the inherent stochasticity of structural inference algorithms, independent structural inference followed by alignment overestimates the difference between data sets and results in irreproducible output. Though there are many Hi–C structural inference methods, ours is the first to jointly model two Hi–C data sets while sharing information between them.

To address the issue of stochasticity, MultiMDS performs MDS embedding and alignment simultaneously on both data sets while minimizing the difference between embeddings multiplied by a similarity weight, which quantifies the degree to which the embeddings are forced to be similar (Algorithm 1). A higher similarity weight results in fewer, higher confidence differences between the estimated structures. The embedding difference term is easily incorporated into the MDS loss function (see “Methods”), another advantage of using MDS instead of PCA, which does not use a loss function.

The stochasticity of independent MDS can be observed when inferring and aligning structures from the same data set across multiple iterations. The root mean square distance (RMSD), which should be zero, is much higher for independent MDS than for MultiMDS run with a similarity weight of 0.02 (Additional file 1: Fig. S1). Next, we tested the ability of MultiMDS to align chromosomal structures from different cell types. We quantified the effects of various similarity weights on the alignment of GM12878 and K562 chr21. A similarity weight of zero is equivalent to independent MDS, so in this condition, we performed alignment after structural inference. We tested the effect of MultiMDS on reproducibility, measured as the correlation between pairwise MultiMDS output across multiple runs with the same input, at similarity weights between 0 and 0.1, to demonstrate that large similarity weights are not needed. Even a similarity weight of 0.05 suffices to improve the reproducibility of alignment to near perfect (Fig. 1c). On the other hand, even large similarity weights do not significantly worsen embedding accuracy relative to independent MDS, as the embedding error (measured as the RMSD between the distance matrix derived from the input Hi–C contact matrix and the distance matrix derived from the embedded structure) for each data set increases little even at a similarity weight of 0.5 (Additional file 1: Fig. S2), suggesting that there are multiple structures that fit the data similarly well. For another example, we aligned mouse embryonic stem cell (mESC) and mouse hepatocyte chr19 with various similarity weights. A similarity weight of 0.04 suffices to improve reproducibility for this comparison (Additional file 1: Fig. S3). In general the optimal similarity weight depends on the data sets being compared and can be inferred by testing reproducibility at a variety of weights. MultiMDS provides the option to use the same partitioning algorithm as miniMDS, an approximation of MDS used for individual structural inference on Hi–C data sets, to improve efficiency [25]. Like miniMDS, MultiMDS is computationally efficient, with little increase in computational time relative to independent structural inference and alignment (Additional file 1: Fig. S4). MultiMDS can be run on very high-resolution data. For example, we ran MultiMDS on GM12878 and K562 at 5-kb resolution, the highest resolution at which Hi–C data was available for multiple mammalian cell types (Additional file 1: Fig. S5). 
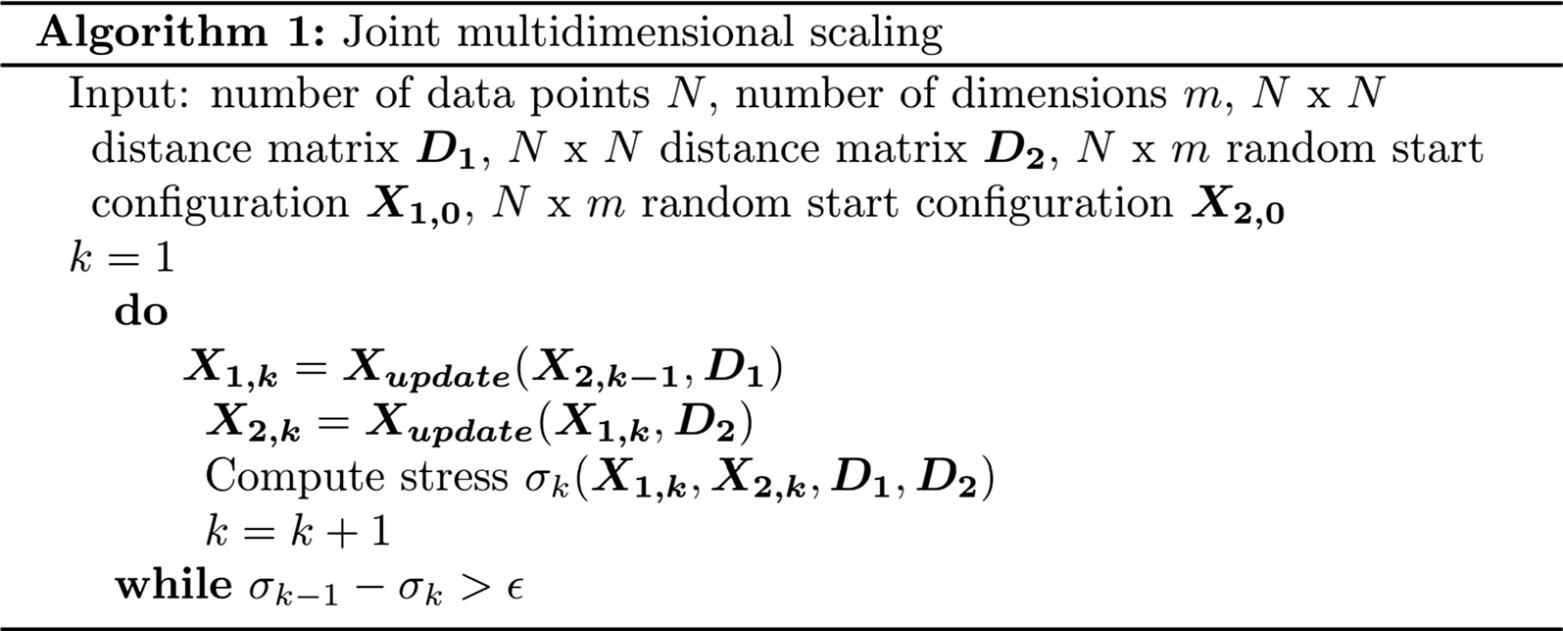


### MultiMDS identifies simulated differential boundaries

We simulated two chr21 Hi–C data sets using sim3C, which probabilistically generates reads based on simulated chromosomal interaction domain (CID) boundaries, similar to TADs [26]. The two data sets differ at one boundary, which is located at 39 Mb in one data set and 40 Mb in the other, but otherwise have identical CIDs (Additional file 1: Fig. S6). However, the locations of particular reads are randomly generated based on the underlying probability distributions, simulating the noise of Hi–C experiments. MultiMDS, but not independent MDS inference and alignment, identified a sharp peak at the differential boundary (Additional file 1: Fig. S7).

### MultiMDS analysis detects known galactose-dependent genomic relocalizations in yeast

To demonstrate the abilities of MultiMDS to align chromosome structures and quantify locus-specific relocalization, we begin with comparisons of yeast intrachromosomal Hi–C data sets. Chromatin structure reorganizes in yeast in response to changes in environment, but this has been difficult to systematically quantify, because yeast do not have A/B compartmentalization to the extent that mammalian cells do. In mammalian cells, long-range chromatin interactions are largely explained by compartment score, which is calculated as the first principal component of the correlation matrix derived from the contact matrix [27]. Compartment score correlates with the position of a locus along an axis between the active nuclear interior (A compartment) and the inactive lamina-associated domains (B compartment) [28] (Additional file 1: Fig. S8). Yeast lack a nuclear lamina [29] and so would not be expected to have A/B compartmentalization. As predicted, PC1 explained far less variance of the Hi–C correlation matrix in yeast compared to mouse and human (Additional file 1: Fig. S9A). We also performed linear support vector regression (SVR) on PC1 scores regressed on 3D coordinates from output structures. SVR finds the 3D axis that explains the most variance in scores and calculates the coefficient of determination *R*^2^, the fraction of variance in scores explained by this axis. The lower *R*^2^ values for compartment scores regressed on yeast structures, relative to mouse and human, suggest that PC1 does not correspond to a single physical axis in yeast (Additional file 1: Fig. S9B).

A previous study compared Hi–C data from yeast grown with glucose to yeast grown with galactose but was limited to measuring differential interaction frequency between pairs of loci, which cannot identify loci that drive changes [30]. Because the experiments were performed in hybrid yeast (*Saccharomyces cerevisiae* × *Saccharomyces uvarum*), it was possible to phase the data by homologs. The Has1–Tda1 locus was shown to pair with its homolog upon galactose induction.

**Fig. 2:**
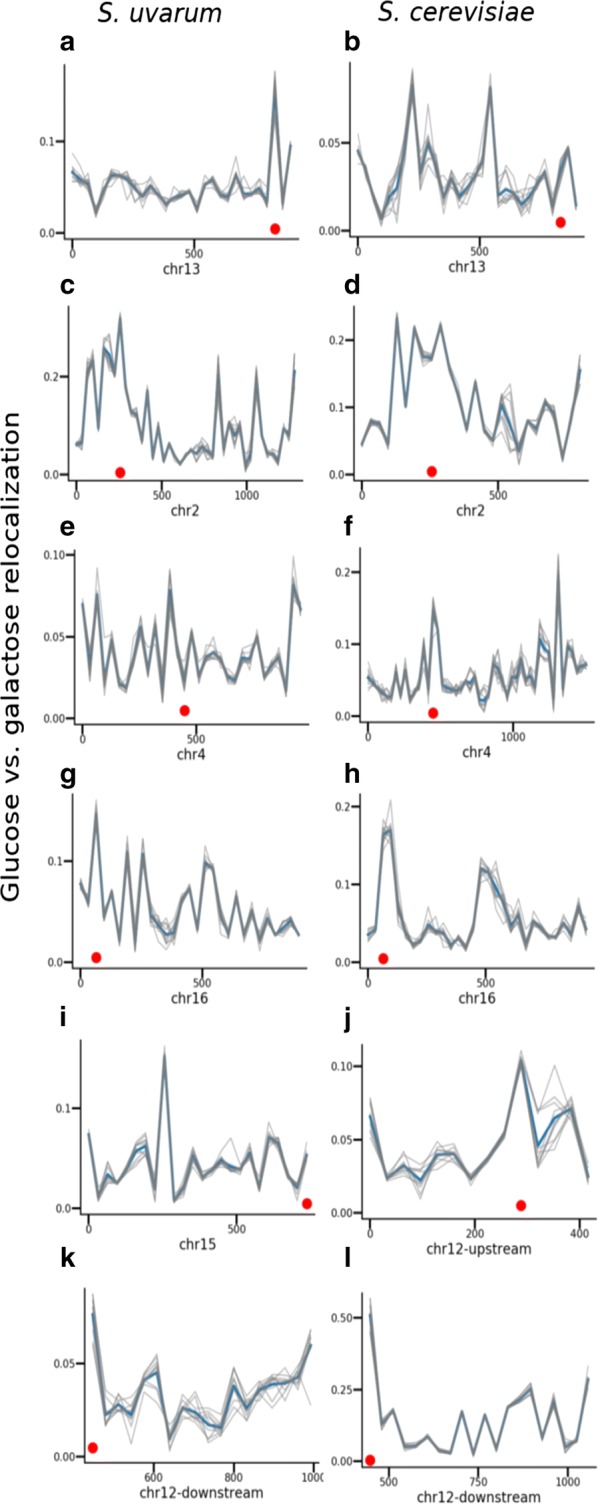
MultiMDS unitless relocalization distance of loci between conditions, for selected chromosomes. Gray: individual iterations. Blue: mean. Genomic coordinates (kb) are shown on *x* axes. **a**, **b** Has1–Tda1. **c**, **d** Gal1–Gal7–Gal10. **e**, **f** Gal3. **g**, **h** Gal4. **i**, **j** Gal2. **k**, **l** rDNA

**Fig. 3:**
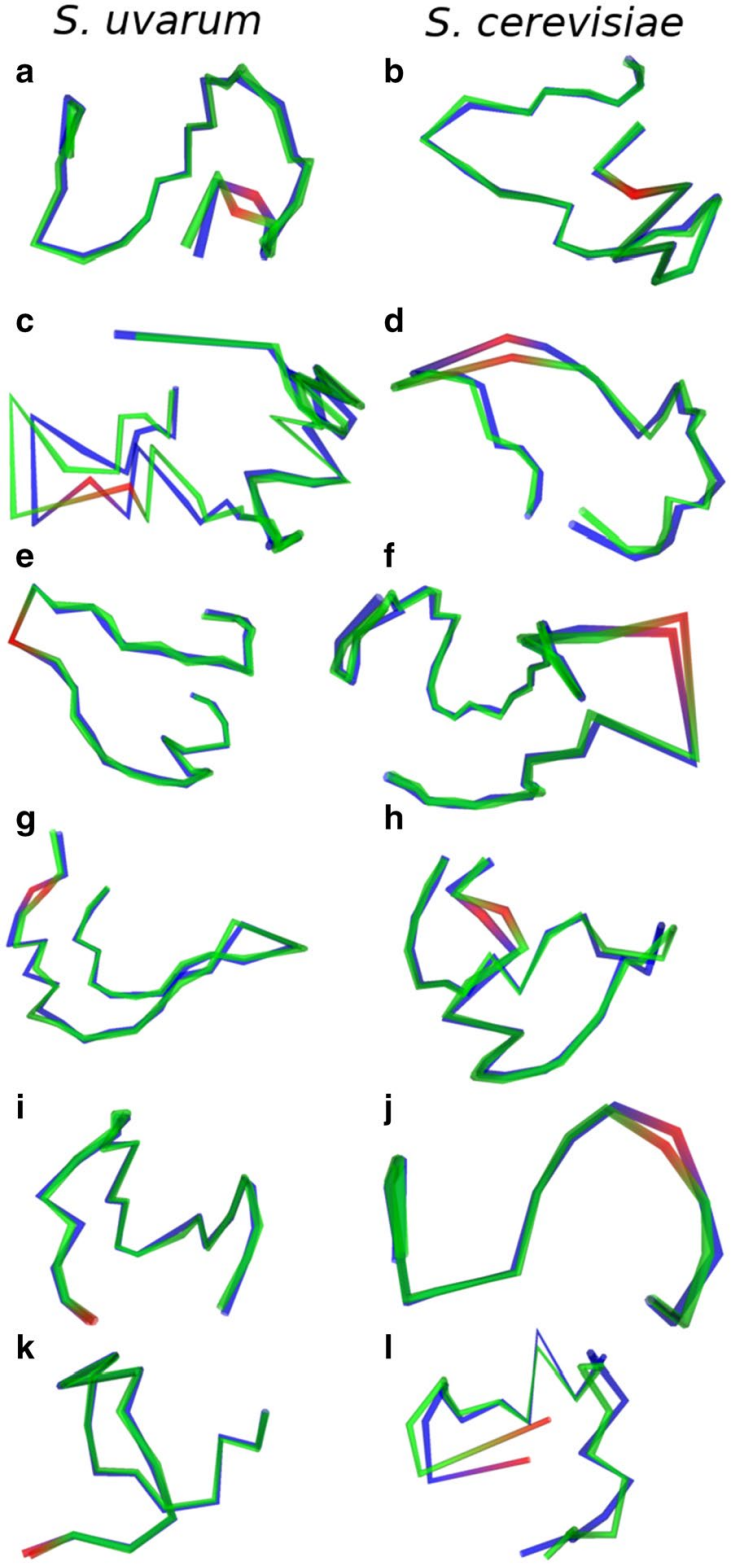
Aligned structures for glucose (blue) and galactose (green) conditions. Loci of interest are highlighted in red, and are presented in the same order as in Fig. 2

MultiMDS comparison between glucose- and galactose-responsive intrachromosomal Hi–C data sets for each yeast chromosome appropriately detects relocalization of the Has1–Tda1 locus, but only for the *S. uvarum* homolog (Figs. 2a, b, 3a, b). To validate the robustness of MultiMDS results, we ran each comparison ten times with random initializations. The locations of peaks were consistent across MultiMDS iterations. The quantification suggests that only one locus drives pairing of the homologs, which could not have been determined by Hi–C loop calling. We also confirmed the expected relocalization of the Gal1–Gal7–Gal10 locus (Figs. 2c, d, 3c, d). MultiMDS showed that Gal3 and Gal4 also relocalize in the presence of galactose (Figs. 2e–h, 3e–h), though the relocalization of these genes had not been reported in the original study. The Gal1–Gal7–Gal10 relocalization was stronger for the *S. uvarum* homolog, whereas the Gal3 relocalization only occurred for the *S. cerevisiae* homolog. The Gal4 relocalization occurred for both homologs.

On chr12, we observed that the rDNA genes create a boundary between the upstream and downstream genomic regions. Because this made chr12 difficult to align, we aligned the two parts of the chromosome separately (Additional file 1: Fig. S10). In the upstream region, which excluded the rDNA genes, we observed relocalization of Gal2, with similar magnitude between homologs (Figs. 2i, j, 3i, j). In the downstream region, the *S. cerevisiae* rDNA genes displayed the strongest relocalization of any locus in the genome (Figs. 2k, l, 3k, l), which may be due to a change in conformation in the nucleolus. It has been previously shown that the yeast nucleolus changes conformation in different media, for example galactose or dextrose [31]. Using independent structure inference and alignment, relocalization at most of these genes cannot be observed and the results are dominated by noise, demonstrating the importance of MultiMDS’s joint structural inference (Additional file 1: Fig. S11). Though other examples of relocalized loci are seen in these data sets, the Gal genes and rDNA are among the highest peaks on their respective chromosomes.

Yeast genes, including Gal1–Gal7–Gal10, relocalize to the nuclear periphery upon activation, where they interact with the nuclear pore components, including Nup60 [30]. Differential ChIP-seq enrichment of Nup60 in the presence of galactose relative to glucose had been qualitatively observed in Gal1–Gal7–Gal10, though not Has1–Tda1 [30]. Using peak calling, we observed Nup60 peaks throughout the gene bodies of Gal1–7–10 (Additional file 1: Figs. S12A, S13A), Gal2 (Additional file 1: Figs. S12B, S13A), Gal3 (Additional file 1: Figs. S12D, S13A), and Tda1 but not Has1 (Additional file 1: Figs. S12C, S13A). Differential enrichment was also found near the transcription start site of Gal4 (Additional file 1: Figs. S12E, S13A). As a negative control we found that Hxt1, a glucose transporter, lost Nup60 binding in the presence of galactose (Additional file 1: Figs. S12F, S13A). Tda1 and all relocalized Gal genes were upregulated, and Has1 and Hxt1 were downregulated (Additional file 1: Fig. S13B).

### Inter-compartment relocalizations dominate mammalian cell-specific differences in genome structure

Despite heterogeneity in chromosome conformation between individual cells, distinct patterns of compartmentalization can be observed between mammalian cell types, which correlate with differences in gene regulation [2, 3]. The detection of compartment changes in population Hi–C data suggests that localization relative to the nuclear periphery, and possibly other landmarks, could be detected using MultiMDS.

We first tested whether MultiMDS was able to identify a consistent axis representing compartmentalization. Compartment scores are not used as input to MultiMDS, so there is no guarantee that the two structures agree on this axis. Indeed, when linear SVR is used to regress compartment scores on the 3D coordinate on unaligned structures, on average only 41% of the variance in compartment scores is explained by a single axis in the structures (Additional file 1: Fig. S14). We performed intrachromosomal MultiMDS on GM12878 and K562 data sets. Because these data sets are not phased by homolog, each alignment represents the average of the two homologs. Next, we performed linear SVR on the compartment scores for each 3D coordinate in the aligned structures. On average, 87% of the variance in compartment scores in the GM12878 and K562 data is explained by the SVR axis, which we refer to as the compartment axis. This supports the hypothesis that compartmentalization represents a single physical axis in the nucleus (Additional file 1: Fig. S14), representing position relative to the nuclear periphery [28]. The high SVR coefficients demonstrate that MultiMDS alignments are capturing consistent features of nuclear organization, rather than superficial similarities between the structures.

As expected, differences along the compartment axis correlate with compartment score differences. For example, the antiviral genes Mx1 and Mx2 have a weaker A compartment score in K562 relative to GM12878, associated with a loss of activity, and can be observed relocalizing along the compartment-associated axis in MultiMDS-aligned intrachromosomal structures for these cell types (Additional file 1: Fig. S15).

**Fig. 4:**
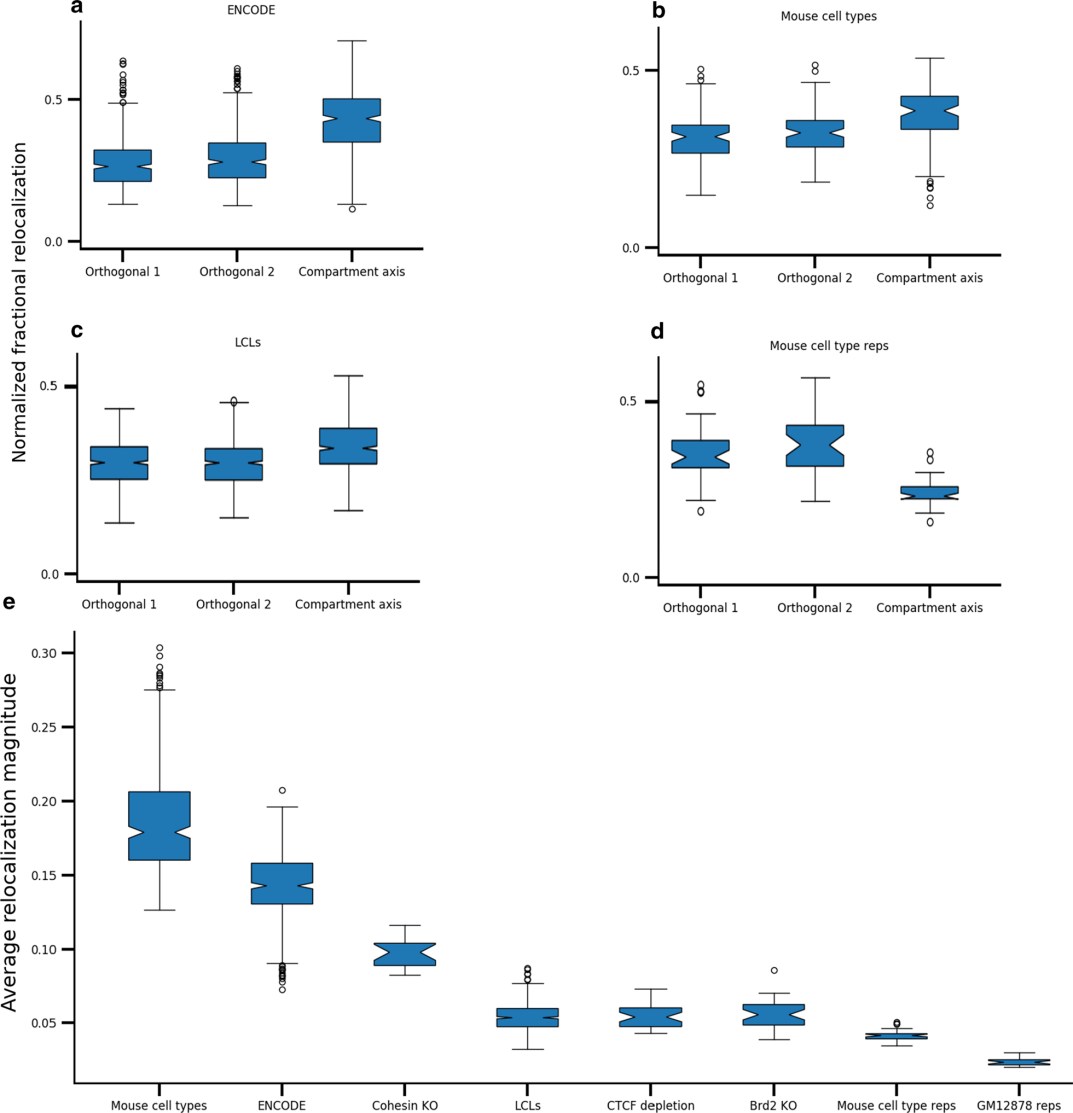
Relocalization distance along each 3D axis as a fraction of total relocalization distance. The compartment axis and two axes orthogonal to it (“Orthogonal 1” and “Orthogonal 2”) are shown. Comparisons are **a** ENCODE cell lines (GM12878, K562, KBM7, HUVEC, HMEC, and NHEK), **b** mouse cell types (G1E-ER4, HPC-7, mESC, and hepatocyte), **c** LCLs, and **d** mouse cell-type replicates. Total magnitude of difference along all axes is shown in **e**

It is clear that cell types differ in compartment score at certain loci, which can be quantified by subtracting normalized compartment scores from different data sets. However, this approach cannot identify compartment-independent differences and thus cannot quantify the extent to which compartment differences explain global differences in Hi–C data. Because MultiMDS relocalization differences are calculated as 3D vectors, they can be decomposed into three components: the difference along the compartment axis, and the differences along the two remaining orthogonal axes (arbitrarily labeled 1 and 2). We can thus calculate the fraction of each locus’s 3D relocalization distance that is attributable to each axis. To exclude the possibility that the physical lengths of each axis confound the results, we divided each fraction by the axis length. We found that the compartment axis is overrepresented for relocalization in pairwise comparisons of ENCODE cell types (GM12878, K562, KBM7, HUVEC, HMEC, and NHEK), even when normalized to axis length (*p* = 2.8 × 10^−149^) (Fig. 4a). Comparisons of mouse cell types (G1E-ER4, HPC-7, mESC, and hepatocyte) revealed a similar magnitude of compartment overrepresentation, indicating that the overrepresentation is not species-specific (*p* < 10^−6^) (Fig. 4b). Compartment axis differences are also overrepresented when comparing lymphoblastoid cell lines (LCLs) from different individuals (*p* = 3.4 × 10^−36^) (Fig. 4c). The overrepresentation of the compartment axis cannot be observed using independent structure inference and alignment (Additional file 1: Fig. S16). The relative overrepresentation of compartment axis relocalizations was consistent regardless of the total magnitude of relocalization, which varied significantly (Fig. 4e).

Given that compartmentalization is highly conserved between replicates (Additional file 1: Fig. S17), we expect relocalizations between replicates to represent noise and not be enriched for compartment differences. Compartment axis overrepresentation was not seen when comparing GM12878 replicates (*p* = 0.08) (Additional file 1: Fig. S18), and the compartment axis was in fact underrepresented in mouse cell-type replicates (*p* = 3 × 10^−9^) (Fig. 4d). The underrepresentation may be due to the compartment axis being more constrained relative to the other axes, which may have more random variation.

**Fig. 5:**
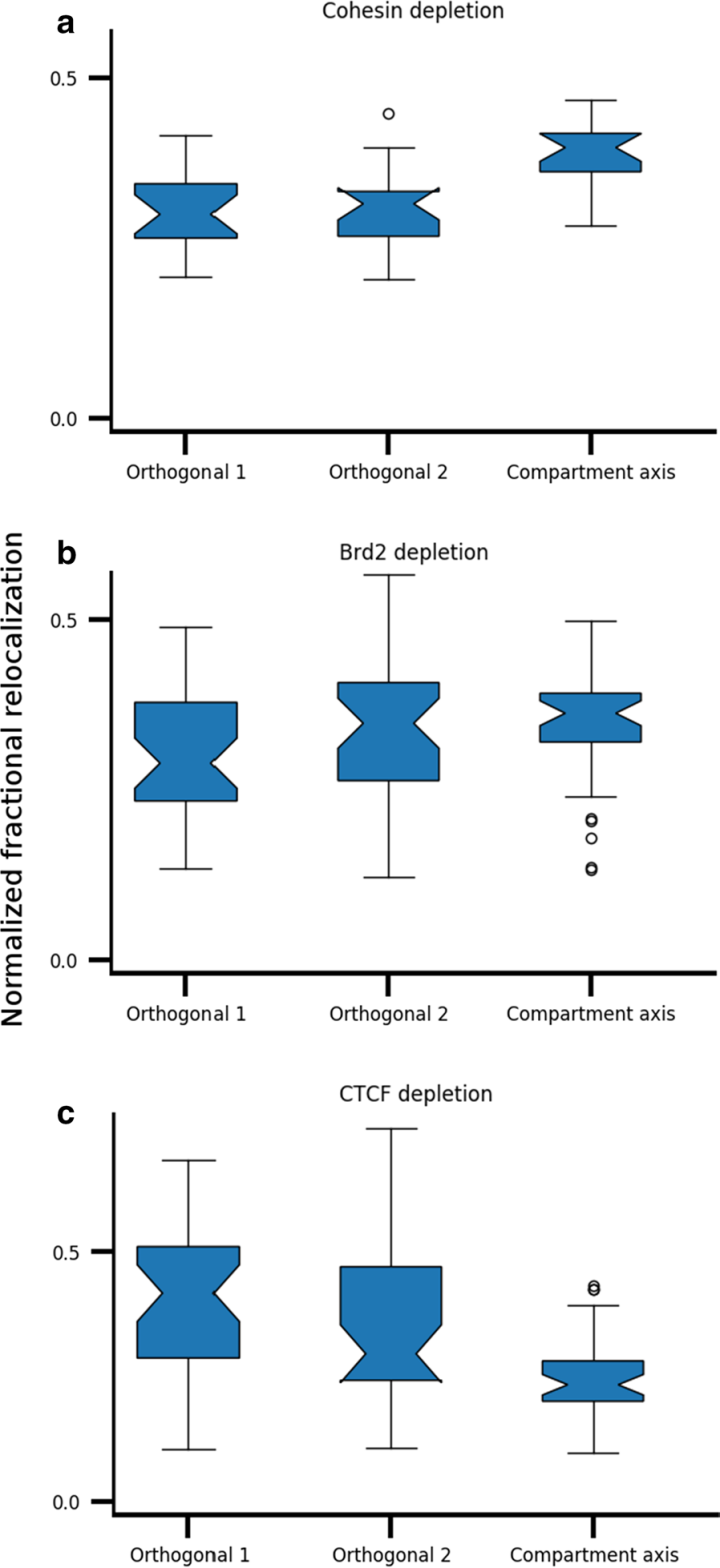
Relocalization distance along each 3D axis as a fraction of total relocalization distance. The compartment axis and two axes orthogonal to it (“Orthogonal 1” and “Orthogonal 2”) are shown. Comparisons are for depletion of **a** cohesin, **b** Brd2, and **c** CTCF

Next we used MultiMDS to validate the relationship between various architectural proteins and compartmentalization. CTCF [17] and Brd2 [18] enforce TAD boundaries, and their loss does not affect compartmentalization. On the other hand, cohesin appears to oppose compartmentalization by forming TADs, and its loss causes a finer-grained compartmentalization to appear [32]. We used MultiMDS to align intrachromosomal Hi–C data sets from wild-type cells to Hi–C data sets resulting from auxin-inducible depletion of CTCF in mESCs, Brd2-knockout (KO) in mouse G1E-ER4 cells, and conditional KO of cohesin loading factor Nipbl in mouse hepatocytes. The relocalizations characterized by MultiMDS in comparisons between cohesin depletion and control Hi–C data are enriched along the compartment axis (*p* < 10^−4^) (Fig. 5a). Conversely, compartment axis relocalizations are not enriched in comparisons between the Brd2 depletion and control Hi–C data (*p* = 0.32) (Fig. 5b) and are slightly depleted in comparisons between the CTCF depletion and controls (*p* < 0.05) (Fig. 5c). The results of MultiMDS serve as validation of previous findings about the role of these proteins in the 3D genome.

### MultiMDS detects intra-compartment relocalizations across cell types

**Fig. 6:**
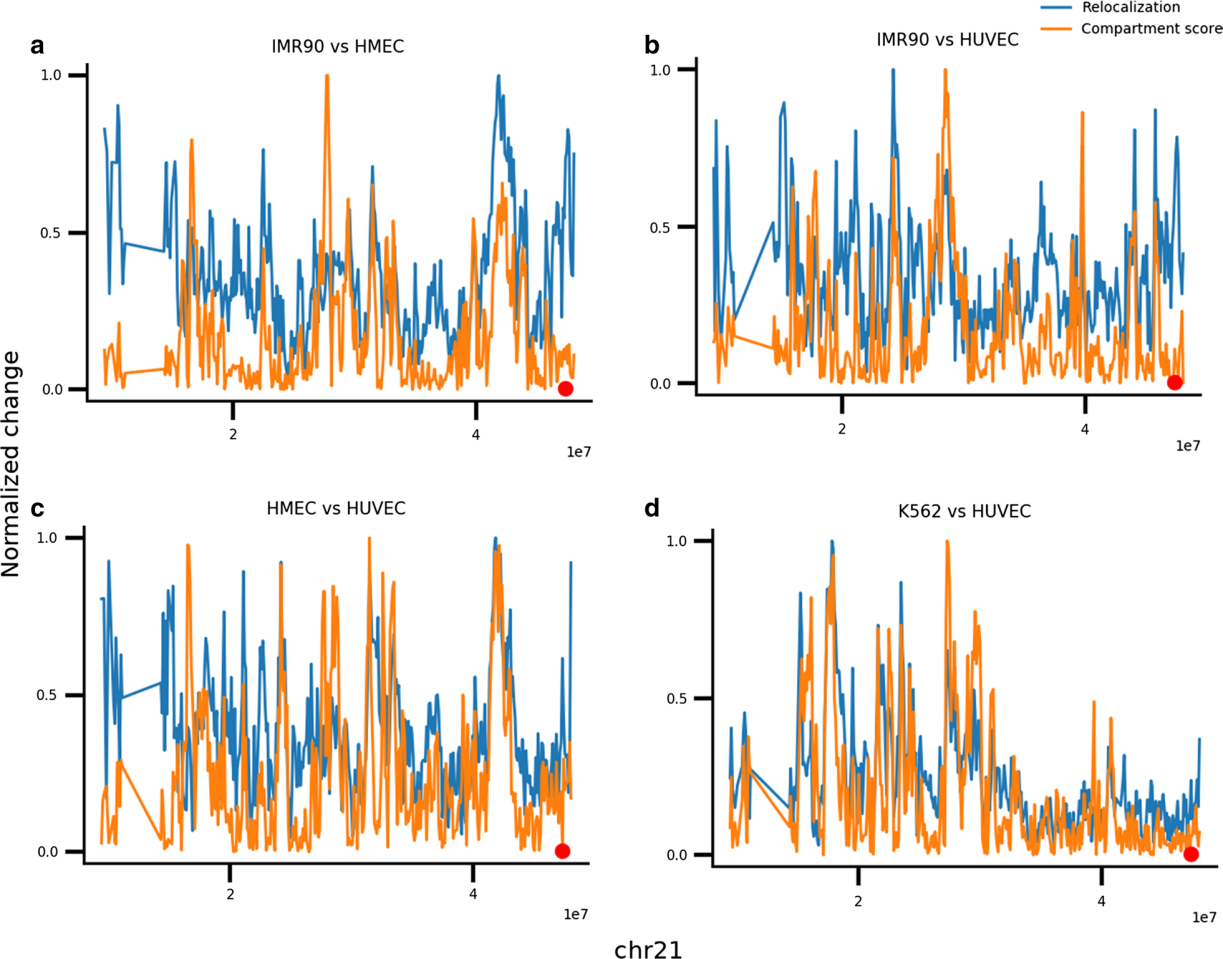
Relocalization and absolute compartment score differences (normalized to 1) between IMR90 and HMEC (**a**), IMR90 and HUVEC (**b**), HMEC and HUVEC (**c**), and K562 and HUVEC (**d**)

Despite the overrepresentation of relocalization along the compartment axis, some orthogonal relocalization also occurs. For example, most relocalization peaks overlap with differential compartment score peaks in comparisons between chr21 structures at 100-kb resolution from IMR90 (embryonic lung fibroblast), HMEC (mammary epithelial), and HUVEC (umbilical vein endothelium) cell lines (Fig. 6a–c). However, some relocalization peaks do not overlap compartment difference peaks. In particular, a relocalization at chr21:47.4–47.5 Mb does not significantly differ in compartment score. This locus contains an intergenic region at chr21:47.75–47.5 Mb that displays different histone modifications across these cell lines (Additional file 1: Fig. S19A). In IMR90 the locus contains several accessible regions displaying H2A.Z, H3K4me1, H3K4me2, and H3K27ac ChIP-seq enrichment, DNase accessibility, and enhancer states (as characterized by the IDEAS genome segmentation approach). HMEC cells also have active marks at this locus, though weaker and without H3K27ac, and have shorter regions of enhancer states, as well as polycomb and heterochromatin states. HUVEC cells have low active ChIP-seq and DNase-seq signals at this locus, with some H3K27me3, and have more polycomb and heterochromatin states and fewer enhancer states. As a negative control, we aligned the HUVEC data with K562 (chronic myelogenous leukemia), a cell line in which this locus has low active ChIP-seq and DNase-seq signal and high H3K27me3 signal and polycomb and heterochromatin states. No relocalization was observed between K562 and HUVEC at this locus (Fig. 6d).

**Fig. 7:**
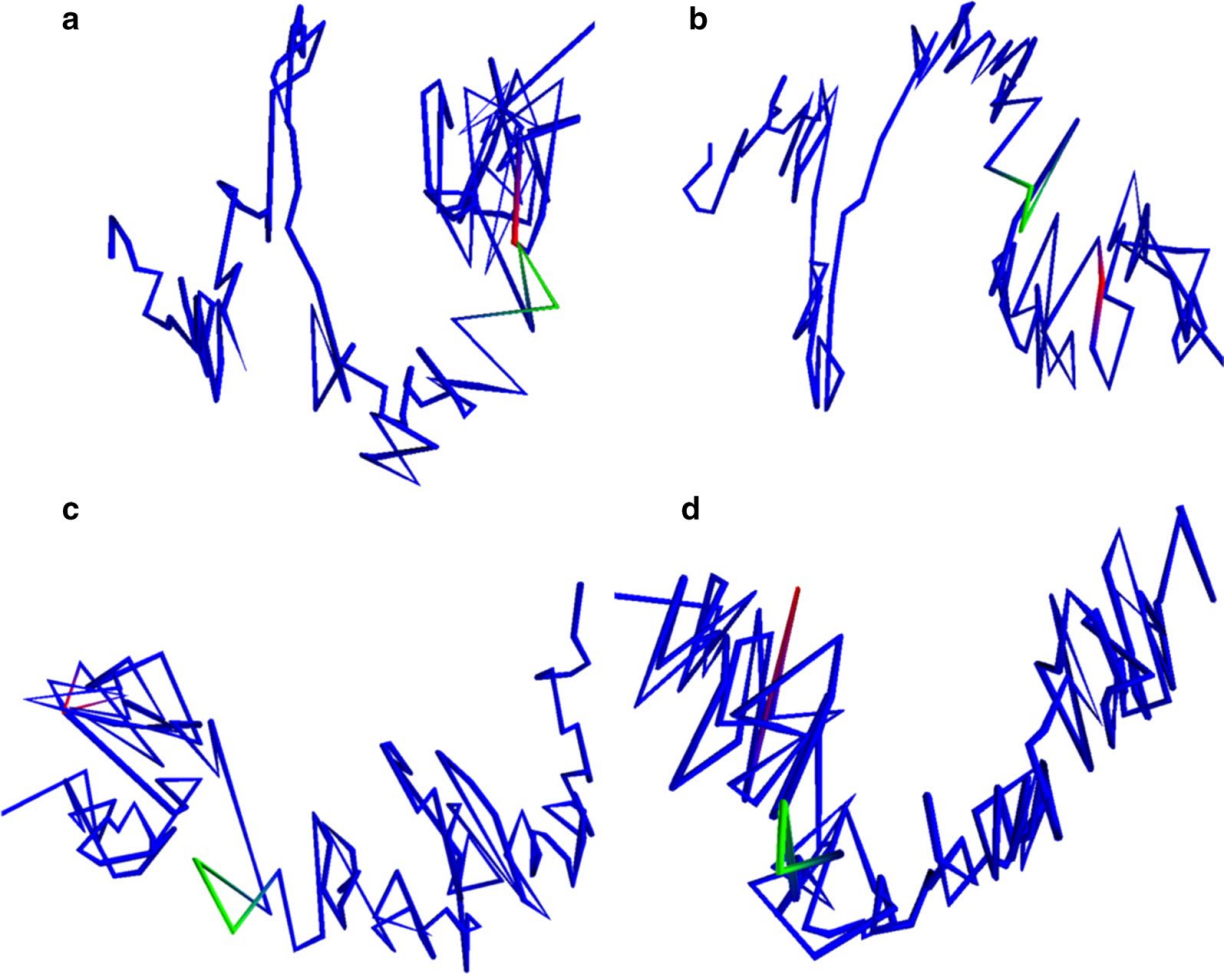
Structures for chr21:45.0–48.1 Mb in IMR90 (**a**), HMEC (**b**), HUVEC (**c**), and K562 (**d**). 47.475–47.5 Mb is highlighted in red, and chr21:46.9–46.975 Mb is highlighted in green

We then performed intrachromosomal MultiMDS alignment at 25-kb resolution to visualize the conformation of the chr21:47.75–47.5 Mb locus. For clarity, we viewed structures for each cell type individually. In IMR90, the locus appears to contact the chr21:46.9–47.0 Mb locus (Fig. 7a). In HMEC, the locus is closer to the chr21:46.9–47.0 Mb locus than in HUVEC and K562, but does not directly contact it (Fig. 7b–d).

**Fig. 8:**
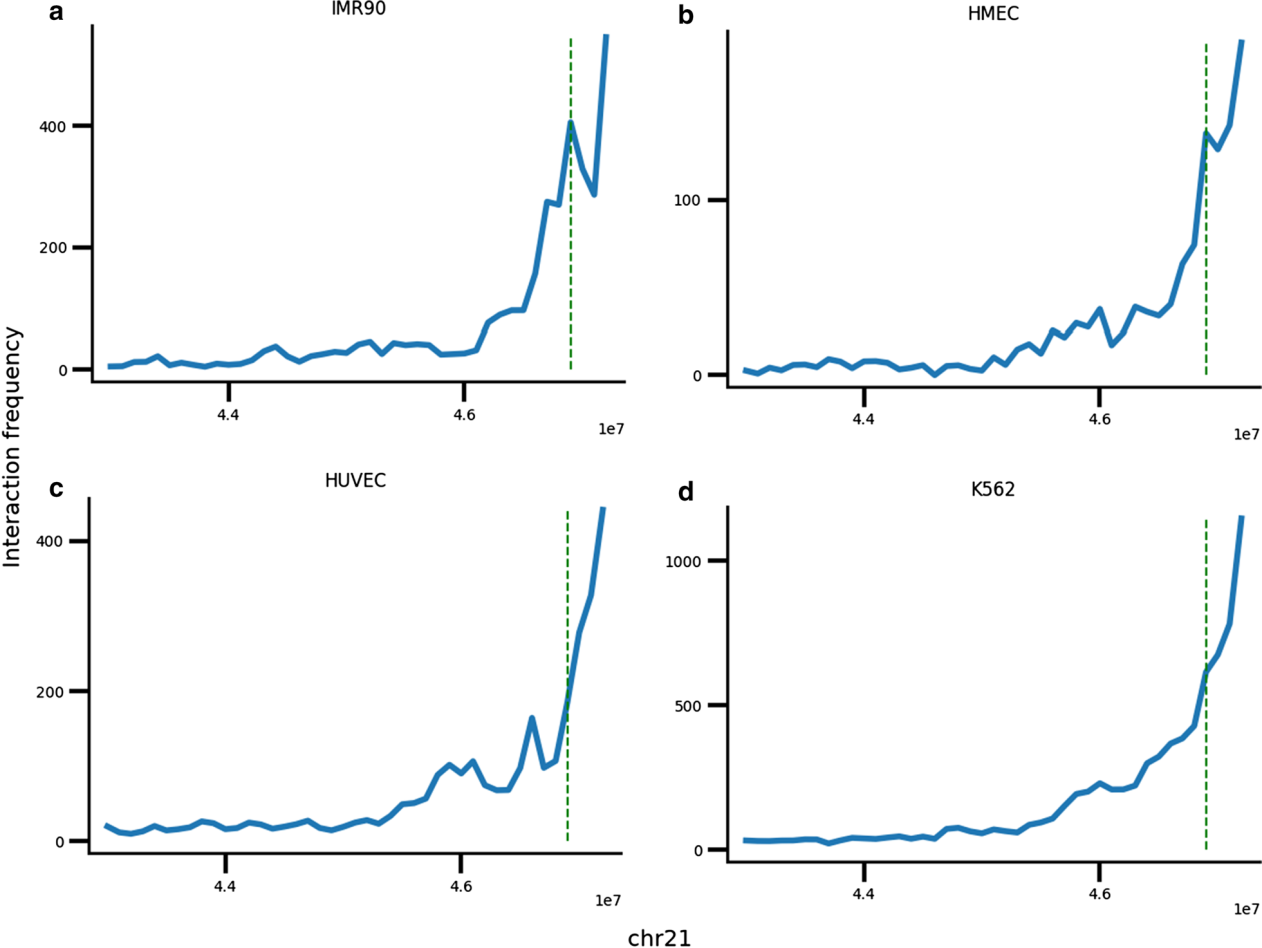
Virtual 4C plots with viewpoint at chr21:47.4–47.5 Mb for IMR90 (**a**), HMEC (**b**), HUVEC (**c**), and K562 (**d**). Green dashed line shows 46.9–47.0 Mb

Virtual 4C plots from the viewpoint of chr21:47.4–47.5 Mb reveal a strong peak at chr21:46.9–47.0 Mb in IMR90, the same region that the putative enhancer appears to contact in the 3D plots (Fig. 8a). This peak is present but weaker in HMEC (Fig. 8b) and not present in HUVEC and K562 (Fig. 8c, d). The chr21:46.9–47.0 Mb locus contains the COL18A1 gene, a component of the extracellular matrix, which has higher H3K36me3 signal in IMR90 relative to the other cell lines (Additional file 1: Fig. S19B).

The differences in activity at the chr21:47.4–47.5 Mb locus cannot be predicted based on compartment scores alone. Though IMR90 has a slightly higher A compartment score at this locus compared to the other cell lines, HUVEC and K562 have higher compartment scores than HMEC, despite having less activity at this locus (Additional file 1: Table S1).

**Fig. 9:**
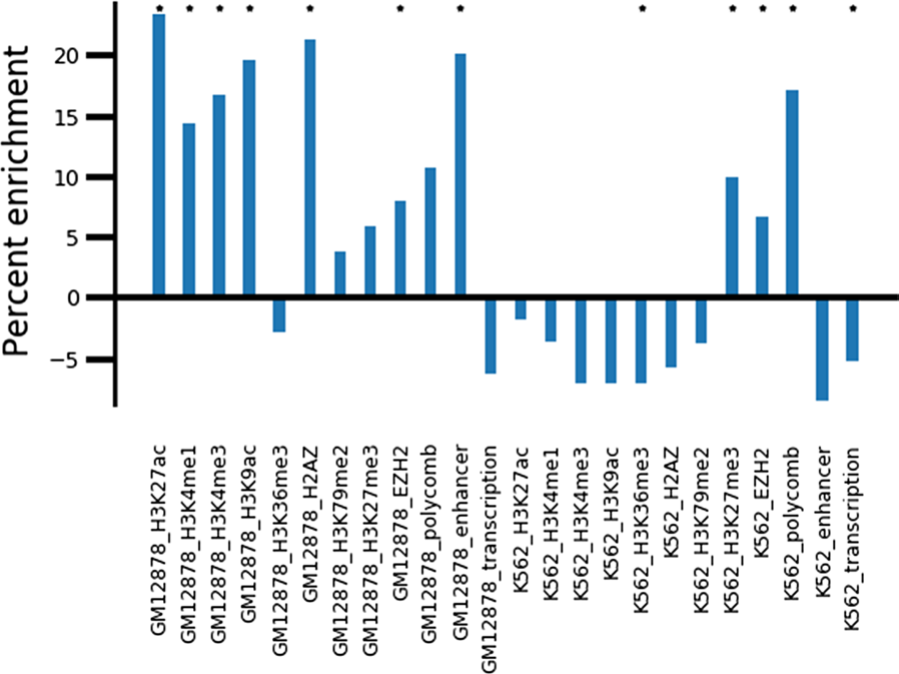
Enrichment of mean coverage of chromatin marks in intra-A relocalization peaks relative to background A compartment. Stars represent *p *< 0.01

Next we performed a genome-wide quantification of intrachromosomal MultiMDS relocalizations with minimal compartment score difference in GM12878 compared to K562. We called peaks in relocalization magnitude for 10-kb bins and, after filtering for mappability, identified peaks with absolute difference in compartment score of less than 0.2 (compartment scores range between − 1 and 1) (Additional file 1: Fig. S20). Though some relocalization peaks do not overlap compartment differences, we noted that few compartment difference peaks occur without relocalization peaks, as would be expected. We analyzed peaks within each compartment separately, so that peaks would not differ from background in compartment composition. We term these intra-A and intra-B relocalization peaks, respectively. Relative to 56,394 mappability-filtered background loci in the A compartment, the 2562 intra-A relocalization peaks were enriched for H3K27ac, H3K4me1, H3K4me3, H3K9ac, H2A.Z, H3K4me2, and IDEAS enhancer states in GM12878, and depleted for these marks in K562 (Fig. 9). The peaks were enriched for the polycomb-associated marks H3K27me3 and EZH2 and IDEAS polycomb states in both GM12878 and K562. H3K36me3 and IDEAS transcription states were depleted in both GM12878 and K562. These differences were not due to the intra-A relocalization peaks differing in compartment score between the cell types or to the peaks having higher compartment scores than the background (Additional file 1: Fig. S21A, B). In fact, the peaks have slightly lower compartment scores than the background, so they would be expected to be depleted of active marks. Most of these results cannot be observed using independent MDS (Additional file 1: Fig. S22A).

The 2543 intra-B relocalization peaks had similar enrichments for histone modifications and states relative to 57,263 mappability-filtered background loci in the B compartment (Additional file 1: Fig. S23). However, the peaks have slightly higher compartment scores than the background (Additional file 1: Fig. S21D), so compartment effects could explain this enrichment.

**Fig. 10:**
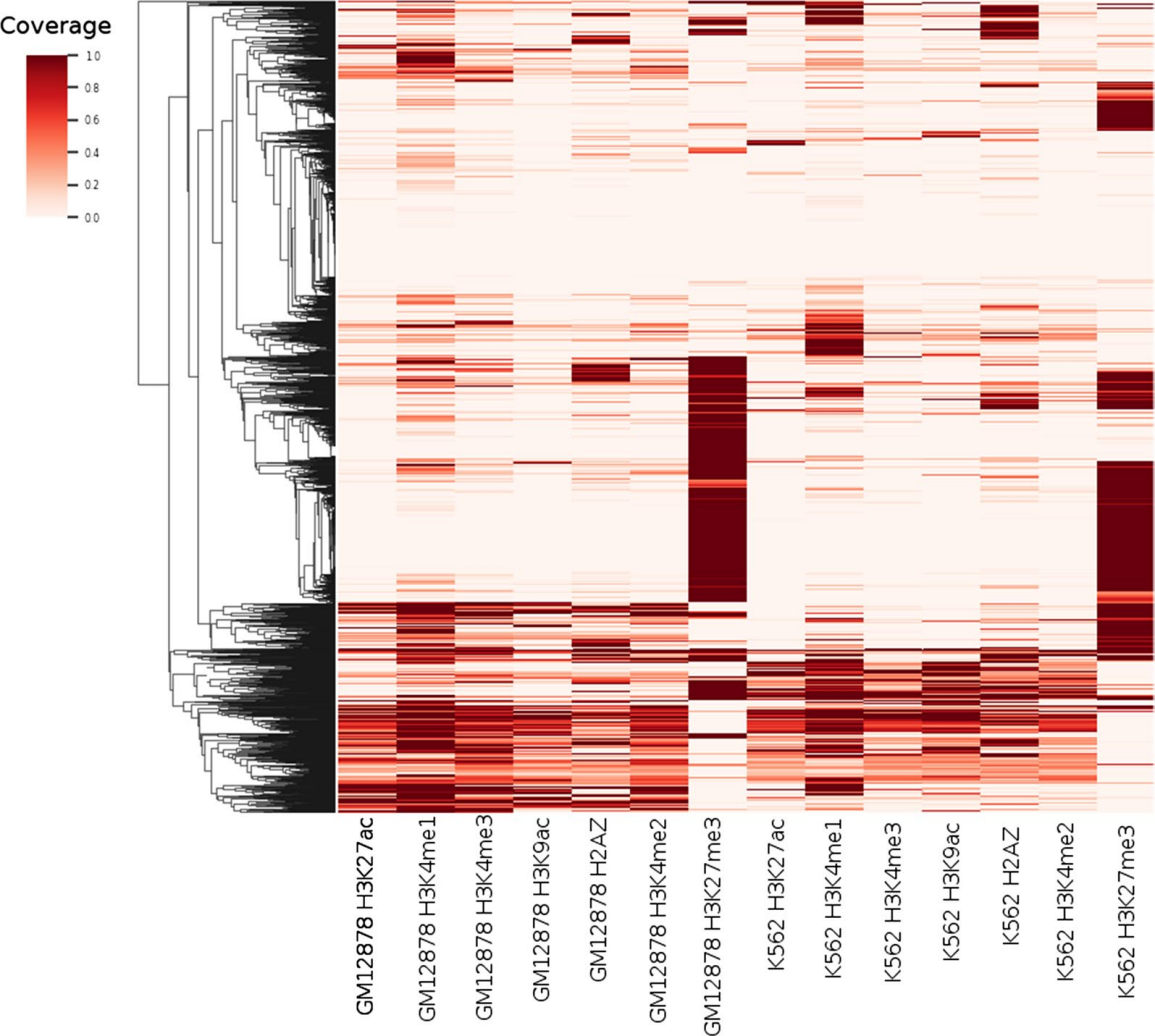
Hierarchical clustering of ChIP-seq peak coverage in intra-A relocalization peaks

Using hierarchical clustering of ChIP-seq coverage, we identified distinct subsets of intra-A relocalization peaks (Fig. 10). A large fraction of peaks are highly enriched for active marks. While some of these peaks are active in both GM12878 and K562, some lose active marks in K562 and gain H3K27me3. Other peaks have H3K27me3 in only one cell type but lack active marks in the other cell type.

In summary, MultiMDS enables the detection of intra-compartment relocalizations, for example loci that change localization between two cell types but remain in the A compartment in both. Such intra-compartment relocalizations appear to be correlated with cell-type-specific changes in regulatory activity.

## Discussion

MultiMDS is a computationally efficient tool for quantifying locus-specific differences between Hi–C data sets, which can be used even for Hi–C data that lacks compartment scores or TAD calls. It captures the known effects of A/B compartment score differences and quantifies the contribution of these differences to global differences in Hi–C data. At the same time, MultiMDS is able to go further than previous types of Hi–C analysis by identifying differences not based on differential compartmentalization, TADs, or looping. MultiMDS also differs from differential loop calling tools, because it can identify the specific locus that drives loop formation. We demonstrated this ability by showing that a single homolog drives galactose-inducible homolog pairing at the Has1–Tda1 locus.

Because of the strong correlation between regulatory activity and compartmentalization, differences in compartment score have dominated previous comparisons of Hi–C data. To explore compartment-independent differences, we used Hi–C data from yeast, which lack compartmentalization. Examples of loci that change conformation upon galactose induction, such as Gal1–Gal7–Gal10 [30] and the nucleolus [31], had been previously identified, but comparisons had not been performed systematically. We confirmed these examples and also showed that Gal2, Gal3, and Gal4 relocalize in response to galactose. All the relocalized Gal genes are upregulated and gain Nup60 peaks, suggesting that the effect occurs via nuclear pore association. It had originally been reported that Has1–Tda1 does not gain Nup60 binding, based on visual inspection, but a quantitative analysis revealed a Nup60 peak at Tda1 upon galactose induction. The gain in nuclear pore association is consistent with the upregulation of Tda1 and its role in glucose metabolism [30].

Due to the strong effect of compartmentalization on long-range interactions, it is challenging to remove the effect of compartment score differences, which are strongly associated with differences in gene regulation, from other differences in chromosomal structure. The contribution of compartment differences to overall structural differences was enriched in both mouse and human cell types and even in LCLs from different individuals. During the preparation of this manuscript, results were published showing that variation in chromosome conformation between LCLs is correlated with gene regulation [33]. The overrepresentation of the compartment axis may be because differences along orthogonal axes are less functional and more stochastic and are thus less visible in aggregate Hi–C data. Consistent with this hypothesis, differences along the compartment axis are depleted in comparisons of mouse cell-type replicate Hi–C data sets. As expected, a large fraction of changes after cohesin depletion are driven by compartment changes, specifically a gain in compartmentalization strength, while Brd2 depletion and CTCF depletion do not significantly affect compartmentalization.

Despite the enrichment of compartment axis relocalizations, we identified examples of loci that relocalized without significant compartment score differences. While it is possible that some compartment-independent relocalizations are driven by compartment-dependent reorganization of distal loci, several display evidence of changes in regulatory activities. For example, a putative enhancer on chr21 relocalizes between cell types in which it appears active, poised, or polycomb repressed, suggesting that these states correspond to three distinct conformations. The presence of a cell-type-specific enhancer at this locus was consistent with the results of our genome-wide quantification of loci that relocalize within the A compartment between GM12878 and K562 with minimal compartment score difference. The relocalized loci were enriched for enhancers and polycomb in GM12878, suggesting active and poised enhancers, but were depleted for enhancers and enriched for polycomb in K562. The contrast between activity and repression may hint at global differences in regulation that occur between K562 and GM12878. On the other hand, the relocalized loci were depleted of active transcription in both cell types relative to the background A compartment. Given the strong relationship between compartment score differences and differential gene expression [2, 3], the correlation with histone modifications further supports the hypothesis that the relocalizations we identify represent a compartment-independent regulatory mechanism.

The enrichment of enhancers at the relocalizations may be due to promoter–enhancer looping, as in the example of the enhancer on chr21. One possibility is that the enhancers are differentially associating with nuclear speckles. It has been shown that distance from nuclear speckles in the A compartment is independent of the nuclear lamina compartment axis and is correlated with super-enhancers and H3K4me3, H3K9ac, and CTCF peaks [34]. Thus the compartment-independent relocalizations we identified may represent differences in nuclear speckle association. Other relocalization peaks have cell-type-specific H3K27me3 without active marks, which may be associated with polycomb hubs that organize the 3D genome [35, 36].

## Conclusions

MultiMDS is a user-friendly tool that provides both visual and quantitative metrics of relocalization, as well as a method for quantifying the contribution of the compartment axis to global differences, which could be used, for example, to determine the role of other architectural proteins in compartmentalization. Though MultiMDS output cannot be interpreted as physical structures, MultiMDS is able to capture consistent structural features present throughout the population of cells. Our preliminary results showing the correlation of functional features with compartment-independent relocalizations hint at the existence of novel forms of nuclear organization, which can be further explored using MultiMDS. As more Hi–C data sets are produced, the number of possible comparisons will increase exponentially, improving our understanding of the relationship between 3D chromosomal structure and function.

## Methods

### Data sets

We used published data sets for all analyses: normalized Hi–C data sets from yeast grown in glucose and galactose media, which were aligned to the sacCer3 reference genome [30]; raw Hi–C counts from ENCODE cell lines, which were aligned to the hg19 reference genome [37]; normalized Hi–C data from lymphoblastoid cell lines (LCLs), which were aligned to the hg38 reference genome [38] made available through the 4D Nucleome Project [39]; ICE-normalized Hi–C data from wild-type and Brd2-knockout (KO) G1E-ER4 cells [18], Hi–C reads from HPC-7 cells [40], ICE-normalized Hi–C data from wild-type and *Nipbl* conditional KO mouse hepatocytes [32], and ICE-normalized Hi–C data from wild-type and CTCF auxin-depleted mouse embryonic stem cells (mESCs) [17]. All mouse data sets were aligned to the mm9 reference genome. Autosomes were used for all analyses. K562 chr9 and chr22 were removed due to their translocation. Hi–C data from ENCODE cell lines was normalized using the provided Knight–Ruiz normalization factors. HPC-7 Hi–C data was normalized by dividing the counts in each bin by the sum of the bin’s row and the sum of the bin’s column. Intrachromosomal MultiMDS was performed for each comparison pair.

Yeast RNA-seq read counts per gene and Nup60 ChIP-seq IP and input reads were from [30]. Nup60 ChIP-seq data was aligned to the sacCer3 reference genome and broad peak calling was performed with MACS2. *Saccharomyces uvarum* gene annotations were from [41]. ChIP-seq data for ENCODE cell lines aligned to the hg19 reference genome was downloaded from http://www.encodeproject.org [42]. Replicated broad peak calls were used for relocalization enrichment analysis, and signal *p* value was used for browser shots. IDEAS 20-state annotation based on Roadmap Epigenomics data was from [43].

### Algorithm

MultiMDS uses a novel joint multidimensional scaling (MDS) algorithm, which simultaneously embeds two distance matrices in a lower dimensional space while minimizing the weighted sum of squared distances (SSD) between the embeddings. MultiMDS incorporates weights representing the expected similarity between the distance matrices, which we refer to as similarity weights. We used equal similarity weights for all loci, equivalent to a single global parameter which is selected empirically for each pairwise comparison (see below).

Assume we have *N* items observed under two conditions. Each condition has an *N* × *N* distance matrix ***D***. The output of our algorithm is two *N* × *m* coordinate matrices ***X***_**1**_ and ***X***_**2**_, where *m* is the number of dimensions of the embedding (3 in our analyses).

The algorithm minimizes stress, which is calculated as

$$\sigma \left( {\varvec{X}_{{\mathbf{1}}} ,\varvec{X}_{{\mathbf{2}}} } \right) = \sum\limits_{i < j} {\left( {d_{ij} (\varvec{X}_{{\mathbf{1}}} ) - \delta_{ij1} } \right)^{2} + \left( {d_{ij} (\varvec{X}_{{\mathbf{2}}} ) - \delta_{ij2} } \right)^{2} } + \sum\limits_{i} {w_{i} d_{i}^{2} (\varvec{X}_{{\mathbf{1}}} ,\varvec{X}_{{\mathbf{2}}} )} .$$

For *c *= 1 or *c *= 2, *d*_*ij*_(***X***_***c***_) is the Euclidean distance between points *i* and *j* in ***X***_***c***_, *δ*_*ijc*_ is the distance between points *i* and *j* from distance matrix ***D***_***c***_, *w*_*i*_ is the similarity weight for point *i*, and *d*_*i*_(***X***_**1**_, ***X***_**2**_) is the Euclidean distance between point *i* in ***D***_**1**_ and point *i* in ***D***_**2**_.

We seek to find a compact expression for SSD, $\sum {w_{i} d_{i}^{2} (\varvec{X}_{{\mathbf{1}}} ,\varvec{X}_{{\mathbf{2}}} )}$, similar to [44]. Consider the weighted squared distance for locus *i*, $w_{i} d_{i}^{2} (\varvec{X}_{{\mathbf{1}}} ,\varvec{X}_{{\mathbf{2}}} )$. Let ***x***_**1*a***_ be column ***a*** of the coordinate matrix ***X***_**1**_, i.e., the *a*th dimension of the embedding. Let *e*_*i*_ be column *i* of the identity matrix ***I***:

$$\begin{aligned} w_{i} d_{i}^{2} (\varvec{X}_{{\mathbf{1}}} ,\varvec{X}_{{\mathbf{2}}} ) & = w_{i} \left[ {\sum\limits_{a = 1}^{m} {\varvec{x}_{{{\mathbf{1}}\varvec{ia}}}^{2} } - 2\sum\limits_{a = 1}^{m} {\varvec{x}_{{{\mathbf{1}}\varvec{ia}}} \varvec{x}_{{{\mathbf{2}}\varvec{ia}}} } + \sum\limits_{a = 1}^{m} {\varvec{x}_{{{\mathbf{2}}\varvec{ia}}}^{2} } } \right] \\ & = w_{i} \left[ {\sum\limits_{a = 1}^{m} {\varvec{x^{\prime}}_{{{\mathbf{1}}\varvec{ia}}} e_{i} e^{\prime}_{i} \varvec{x}_{{{\mathbf{1}}\varvec{ia}}} } - 2\sum\limits_{a = 1}^{m} {\varvec{x^{\prime}}_{{{\mathbf{1}}\varvec{ia}}} e_{i} e^{\prime}_{i} \varvec{x}_{{{\mathbf{2}}\varvec{ia}}} } + \sum\limits_{a = 1}^{m} {\varvec{x^{\prime}}_{{{\mathbf{2}}\varvec{ia}}} e_{i} e^{\prime}_{i} \varvec{x}_{{{\mathbf{2}}\varvec{ia}}} } } \right] \\ & = w_{i} \left[ {\sum\limits_{a = 1}^{m} {\varvec{x^{\prime}}_{{{\mathbf{1}}\varvec{ia}}} \varvec{E}_{\varvec{i}} \varvec{x}_{{{\mathbf{1}}\varvec{ia}}} } - 2\sum\limits_{a = 1}^{m} {\varvec{x^{\prime}}_{{{\mathbf{1}}\varvec{ia}}} \varvec{E}_{\varvec{i}} \varvec{x}_{{{\mathbf{2}}\varvec{ia}}} } + \sum\limits_{a = 1}^{m} {\varvec{x^{\prime}}_{{{\mathbf{2}}\varvec{ia}}} \varvec{E}_{\varvec{i}} \varvec{x}_{{{\mathbf{2}}\varvec{ia}}} } } \right] \\ & = w_{i} \left[ {{\mathbf{Tr}}\varvec{X^{\prime}}_{{{\mathbf{1}}\varvec{i}}} \varvec{E}_{\varvec{i}} \varvec{X}_{{{\mathbf{1}}\varvec{i}}} - 2{\mathbf{Tr}}\varvec{X^{\prime}}_{{{\mathbf{1}}\varvec{i}}} \varvec{E}_{\varvec{i}} \varvec{X}_{{{\mathbf{2}}\varvec{i}}} + {\mathbf{Tr}}\varvec{X^{\prime}}_{{{\mathbf{2}}\varvec{i}}} \varvec{E}_{\varvec{i}} \varvec{X}_{{{\mathbf{2}}\varvec{i}}} } \right], \\ \end{aligned}$$

where ***E***_***i***_ is a matrix with *e*_*ii*_= 1 and all other elements zero.

$$\sum {w_{i} d_{i}^{2} (\varvec{X}_{{\mathbf{1}}} ,\varvec{X}_{{\mathbf{2}}} )} = {\mathbf{Tr}}\varvec{X^{\prime}}_{{\mathbf{1}}} \varvec{WX}_{{\mathbf{1}}} - 2{\mathbf{Tr}}\varvec{X^{\prime}}_{{\mathbf{1}}} \varvec{WX}_{{\mathbf{2}}} + {\mathbf{Tr}}\varvec{X^{\prime}}_{{\mathbf{2}}} \varvec{WX}_{{\mathbf{2}}} ,$$

where ***W*** is a matrix with *w*_*ii*_= *w*_*i*_ and all other elements zero.

Combining with Eq. 8.27 in Borg and Groenen gives

$$\begin{aligned} & \sigma \left( {\varvec{X}_{{\mathbf{1}}} ,\varvec{X}_{{\mathbf{2}}} } \right) \le \eta_{\delta 1}^{2} + {\mathbf{Tr}}\varvec{X^{\prime}}_{{\mathbf{1}}} \varvec{X}_{{\mathbf{1}}} - 2{\mathbf{Tr}}\varvec{X^{\prime}}_{{\mathbf{1}}} \varvec{B}(\varvec{Z}_{{\mathbf{1}}} )\varvec{Z}_{{\mathbf{1}}} + \eta_{\delta 2}^{2} + {\mathbf{Tr}}\varvec{X^{\prime}}_{{\mathbf{2}}} \varvec{X}_{{\mathbf{2}}} \\ & - 2{\mathbf{Tr}}\varvec{X^{\prime}}_{{\mathbf{2}}} \varvec{B}(\varvec{Z}_{{\mathbf{2}}} )\varvec{Z}_{{\mathbf{2}}} + {\mathbf{Tr}}\varvec{X^{\prime}}_{{\mathbf{1}}} \varvec{WX}_{{\mathbf{1}}} - 2{\mathbf{Tr}}\varvec{X^{\prime}}_{{\mathbf{1}}} \varvec{WX}_{{\mathbf{2}}} + {\mathbf{Tr}}\varvec{X^{\prime}}_{{\mathbf{2}}} \varvec{WX}_{{\mathbf{2}}} = \tau \left( {\varvec{X}_{{\mathbf{1}}} ,\varvec{Z}} \right). \\ \end{aligned}$$

*τ*(***X***_**1**_, ***X***_**2**_) achieves its minimum when $\nabla \tau (\varvec{X}_{{\mathbf{1}}} ,\varvec{Z}) = 0$. Holding ***X***_**2**_ constant, we calculate the gradient and solve the system of linear equations as follows:

$$\begin{aligned} & 2\varvec{X}_{{\mathbf{1}}} - 2\varvec{B}(\varvec{Z})\varvec{Z + }2\varvec{WX}_{{\mathbf{1}}} - 2\varvec{WX}_{{\mathbf{2}}} = 0 \\ & (\varvec{W} + \varvec{I})\varvec{X}_{{\mathbf{1}}} = \varvec{WX}_{{\mathbf{2}}} + \varvec{B}(\varvec{Z})\varvec{Z} \\ & \varvec{X}_{\text{update}} = (\varvec{W} + \varvec{I})^{ + } \left[ {\varvec{WX}_{{\mathbf{2}}} + \varvec{B}(\varvec{Z})\varvec{Z}} \right]. \\ \end{aligned}$$

This is the update formula for ***X***_**1**_, where ***W***^+^ is the Moore–Penrose inverse of ***W***. The update formula for ***X***_**2**_ is calculated similarly. The algorithm alternately updates ***X***_**1**_ and ***X***_**2**_ using the coordinates calculated at the previous step.

### Implementation

MultiMDS was implemented in python as a modification of miniMDS [25]. The joint MDS algorithm was implemented using a modification of MDS from scikit-learn [45]. Hi–C data must be normalized to correct for biases prior to using MultiMDS. In addition, MultiMDS normalizes each data set by dividing by its mean, so that the data sets will approximately have the same scale. Loci with zero counts in both data sets are excluded. By default, distances are calculated as (contact frequency)^−¼^ [46], but MultiMDS allows this conversion factor to be changed by users. Contact frequencies of zero were converted to distances of zero, which are ignored by the MDS algorithm. MultiMDS also supports interchromosomal inference and alignment.

MultiMDS incorporates a small distance decay prior to reduce noise. The corrected contact frequency is calculated as $c_{\text{corrected}} = c_{\text{observed}} *(1 - k) + c_{\text{expected}} *k,$, where *c*_observed_ is the observed contact frequency, *c*_expected_ is the average contact frequency for loci pairs with the same linear separation, and *k* is a weight parameter. *k* is set to 0.05 by default, because we have found that this is the smallest value that can consistently produce visually interpretable structures. Without this prior, structures appear as random tangles (Additional file 1: Fig. S24).

### Parameter selection

Similarity weights were selected by identifying the value at which increasing the similarity weight does not further increase reproducibility (see Fig. 1c).

### Comparison to independent structural inference and alignment

To compare MultiMDS with an alternate approach that aligns independently estimated genome structures, we first performed structural inference using miniMDS on each Hi–C data set independently. The two structures were then aligned using the Kabsch algorithm [24], which minimizes the RMSD between two static structures.

### Hi–C simulation

sim3C was used to simulate 150-bp HindIII Hi–C reads from hg19 chr21 (linear). Because sim3C randomly generates CIDs, we modified the code to use custom nonrandom CIDs: chromosome start to 20 Mb, 20 to 39/40 Mb, and 40 Mb to chromosome end. Modified code is available on GitHub.

### Compartment analysis

Compartment scores were calculated as described by [27]. Briefly, a correlation matrix was calculated from the observed/expected Hi–C contact matrix. Compartment scores were defined as PC1 of the correlation matrix. PC1 accounts for most of the variance in the correlation matrix (up to 90% in high-coverage data sets). Scores were normalized to range between − 1 and 1. The active compartment was defined as the compartment with greater enrichment for the IDEAS states 10_TssA, 14_TssWk, 8_TssAFlnk, 17_EnhGA, 6_EnhG, 4_Enh, 5_Tx, and 2_TxWk [43]. The compartment axis for each chromosome was identified using linear support vector regression implemented in scikit-learn with default parameters [45].

To prevent bias due to some axes being physically longer, the fraction of relocalization occurring along each axis was normalized by dividing by the axis length, which was calculated as the average distance of each coordinate from the centroid along that axis. Differences in normalized fractional relocalization were calculated using a two-sided independent-sample *t* test.

### Relocalization peak analysis

Peaks in relocalization magnitude were called using a continuous wavelet transform (CWT) [47], with peak widths ranging from 1 to 10. A CWT identifies peaks in noisy data based on their characteristic shape, without requiring smoothing. Relocalization peaks with absolute compartment score differences of less than 0.2 were overlapped with ChIP-seq peaks and IDEAS states using bedtools to calculate coverage [48], and *p* values were calculated using a two-sided independent sample *t* test. The IDEAS states 17_EnhGA, 6_EnhG, 19_Enh/ReprPC, 18_Enh/Het, 4_Enh, and 11_EnhBiv were called enhancers, 16_TxRepr, 5_Tx, and 2_TxWk were called transcribed, and 13_ReprPC, 12_Het/ReprPC, and 1_ReprPCWk were called polycomb repressed.

### Differential ChIP-seq peaks

MACS2 [49] was used to call broad peaks from Nup60 ChIP-seq data under glucose and galactose conditions separately. Glucose and galactose peaks were merged, and edgeR [50] was run on the tag counts in each peak to identify differential enrichment between conditions. Because replicates were not available, the biological coefficient of variation was estimated as 0.1.

## Supplementary information

**Additional file 1.** Supplementary Figures S1–S24 and Supplementary Table 1.

## Data Availability

The scripts to reproduce figures and analysis are available at https://github.com/seqcode/multimds.

## Abbreviations

CID: chromosomal interaction domain
CWT: continuous wavelet transform
kb: kilobase
KO: knockout
LCL: lymphoblastoid cell line
Mb: megabase
MDS: multidimensional scaling
mESC: mouse embryonic stem cell
PCA: principal component analysis
RMSD: root mean square distance
SSD: sum of squared distances
SVR: support vector regression
TAD: topologically associated domain
