# Supplementary material for: Joint inference and alignment of genome structures enables characterization of compartment-independent reorganization across cell types
Source: Epigenetics Chromatin. 2019 Oct 8;12:61. doi: 10.1186/s13072-019-0308-3 (PMC6784335; doi:10.1186/s13072-019-0308-3)
Supplement: Supplementary file 1 — Additional file 1. Supplementary Figures S1–S24 and Supplementary Table 1. [file 13072_2019_308_MOESM1_ESM.docx]

**SUPPLEMENTARY FIGURES**

**
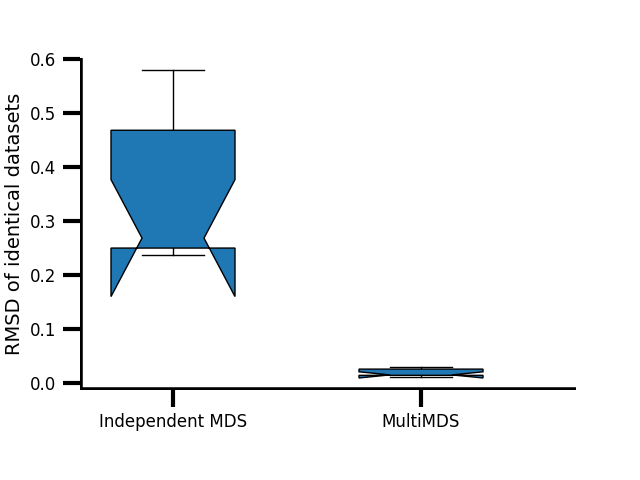
**

**Supplementary Figure 1.** Root mean square distance (RMSD) of aligned structures from identical datasets (GM12878 chr21, 100-kb resolution) independently inferred and aligned from multiple iterations of MDS (left) and jointly inferred and aligned from MultiMDS (right). Each algorithm was tested 10 times.

**
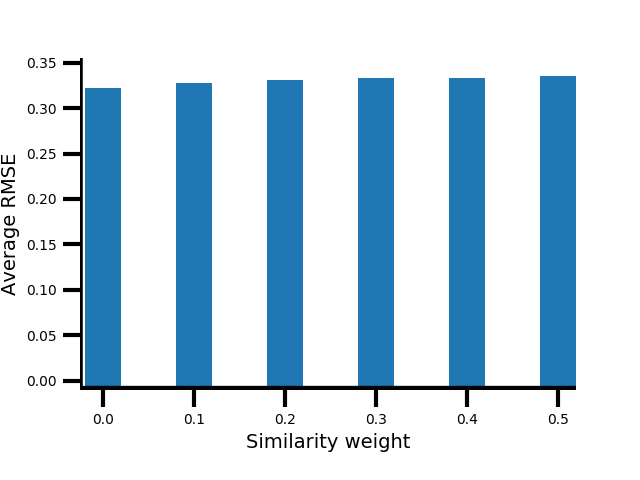
**

**Supplementary Figure 2.** Embedding error for MultiMDS run on GM12878 and K562 chr21, measured across a range of similarity weights.


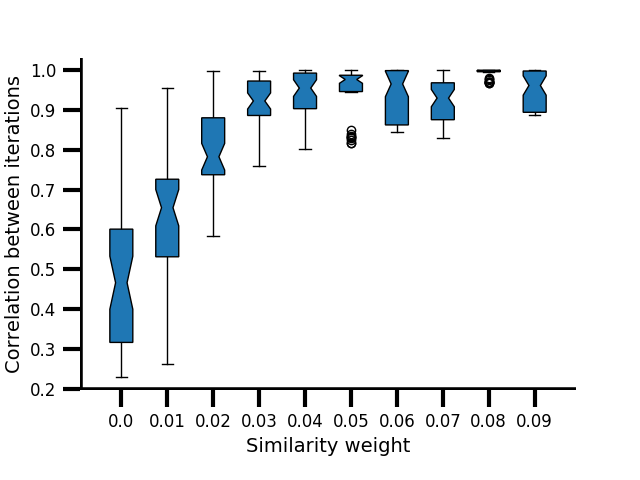


**Supplementary Figure 3.** Pairwise correlations between multiple runs of MultiMDS applied to mESC and mouse hepatocyte chr19, measured across a range of similarity weights. Zero weight represents independent inference and alignment.


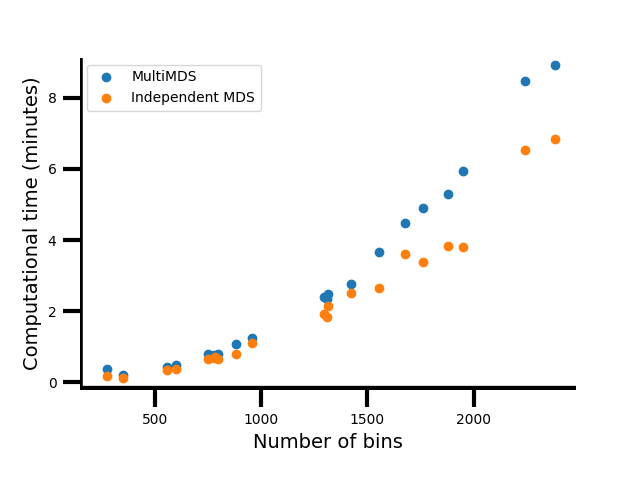


**Supplementary Figure 4.** Computational time for intrachromosomal datasets of various sizes, measured for MultiMDS and for independent MDS structural inference followed by alignment.


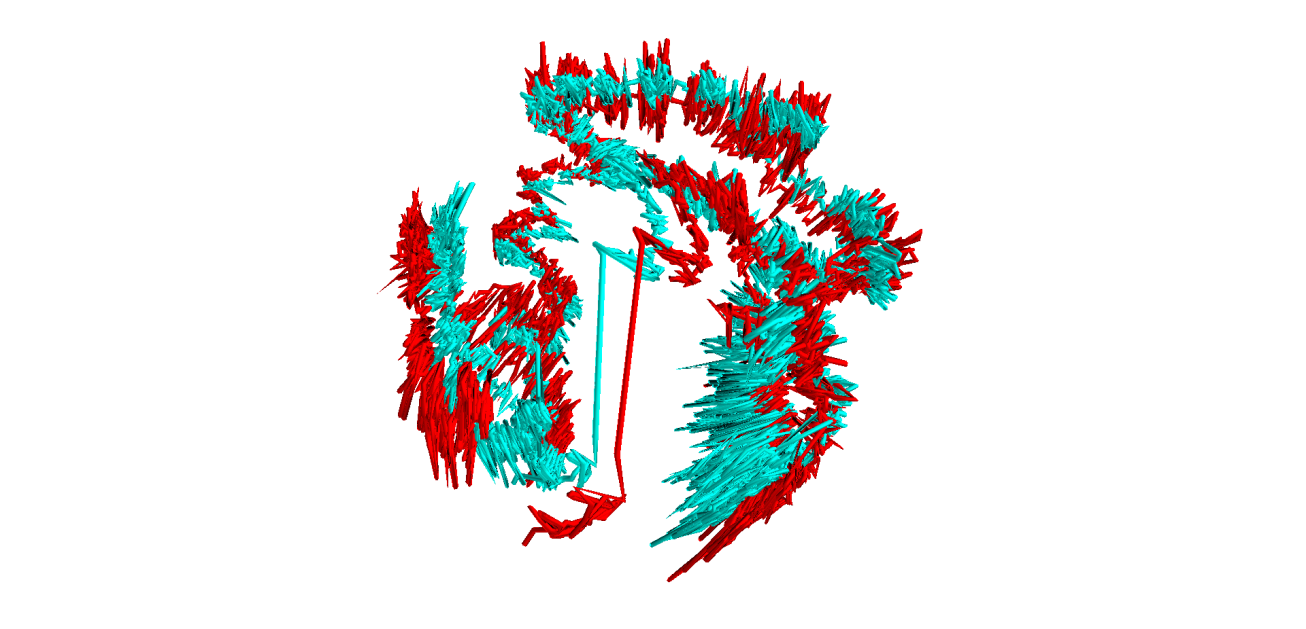


**Supplementary Figure 5.** MultiMDS applied to GM12878 (red) and K562 (cyan) chr21 datasets at 5-kb resolution. Only loci with average mappability > 0.75 are shown.


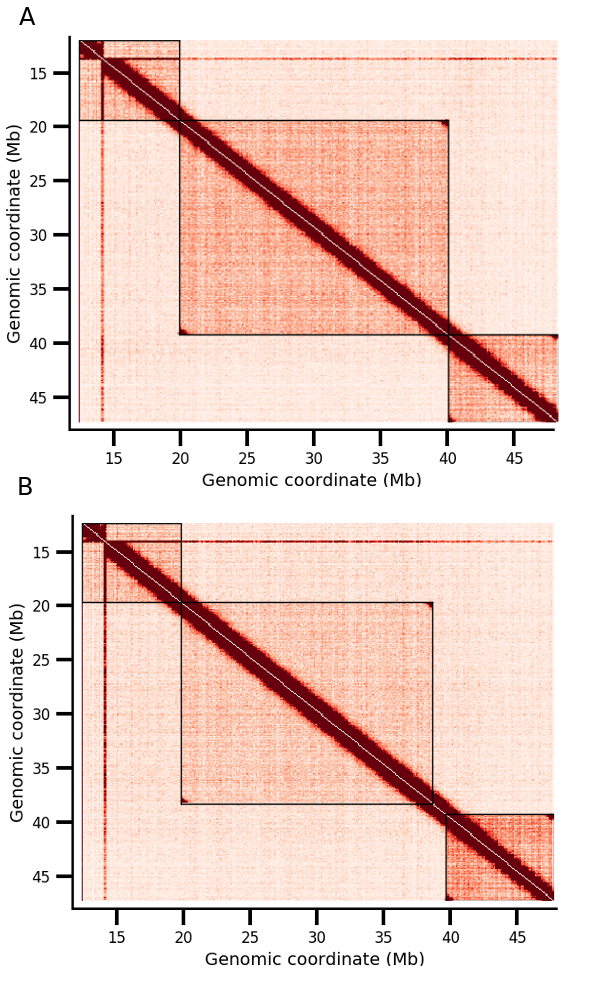


**Supplementary Figure 6.** Simulated chr21 Hi-C contact heatmaps. Black boxes are shown around CIDs. A simulated differential CID boundary is found at 40 Mb in A and 39 Mb at B.


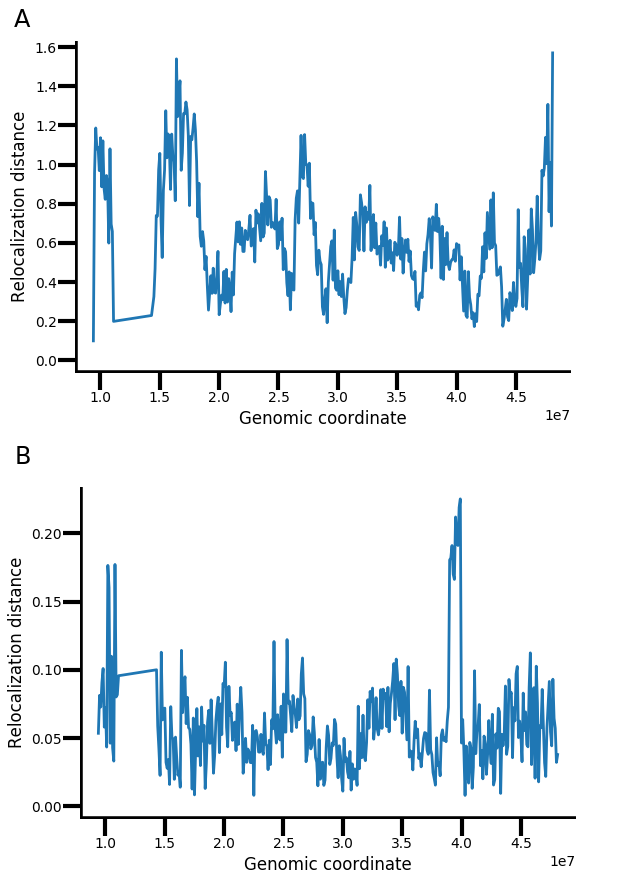


**Supplementary Figure 7.** Relocalization between two simulated datasets calculated with independent MDS inference and alignment (A) and MultiMDS (B).


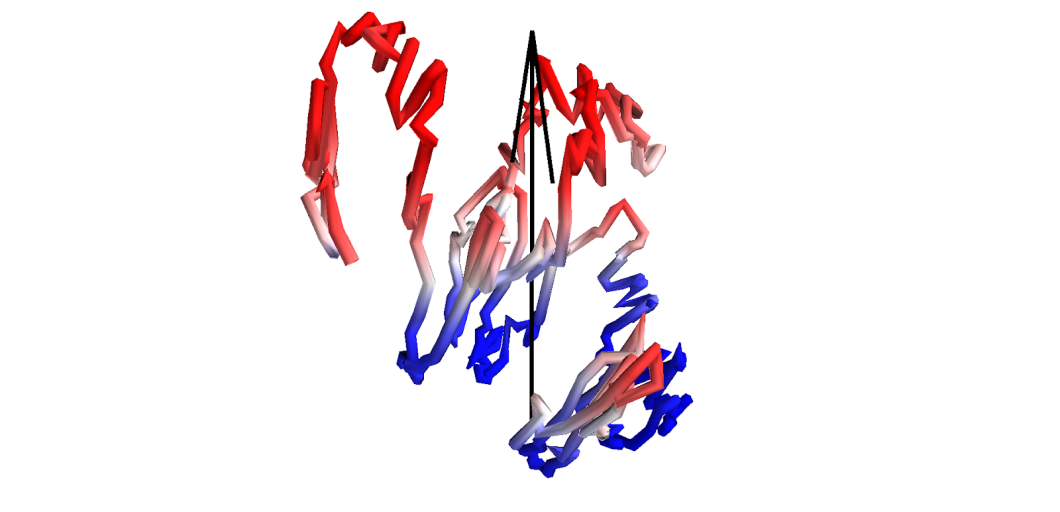


**Supplementary Figure 8.** Compartment score plotted on GM12878 chr21 at 100-kb resolution. Positive scores (corresponding to A compartment) are plotted in reds, and negative scores (corresponding to B compartment) are plotted in blues. The structure has been rotated so that the SVR axis (arrow) aligns with the z axis. Pericentromeric regions have been removed.


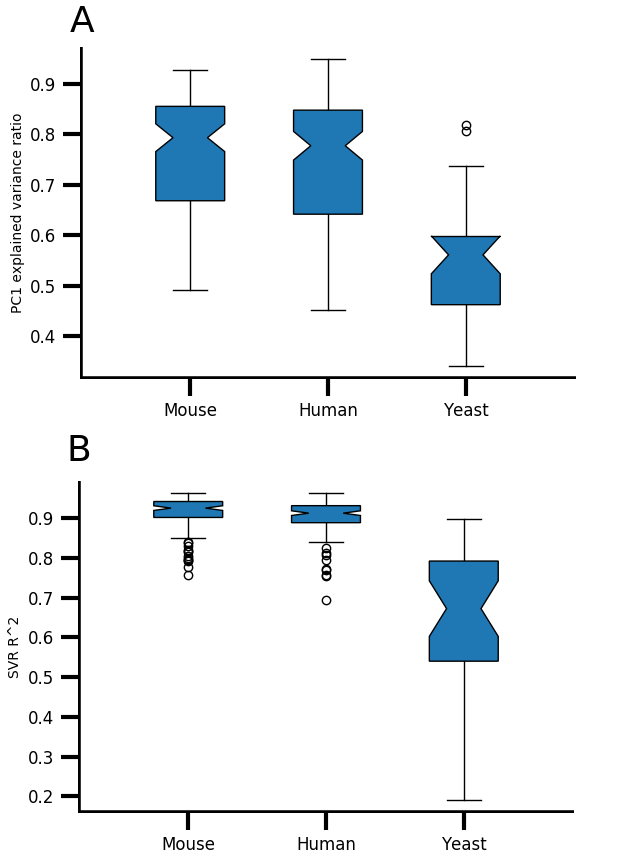


**Supplementary Figure 9. A)** Variance explained by PC1 of Hi-C correlation matrix by species. **B)** Linear SVR coefficient of determination of compartment scores predicted by 3D coordinates by species.


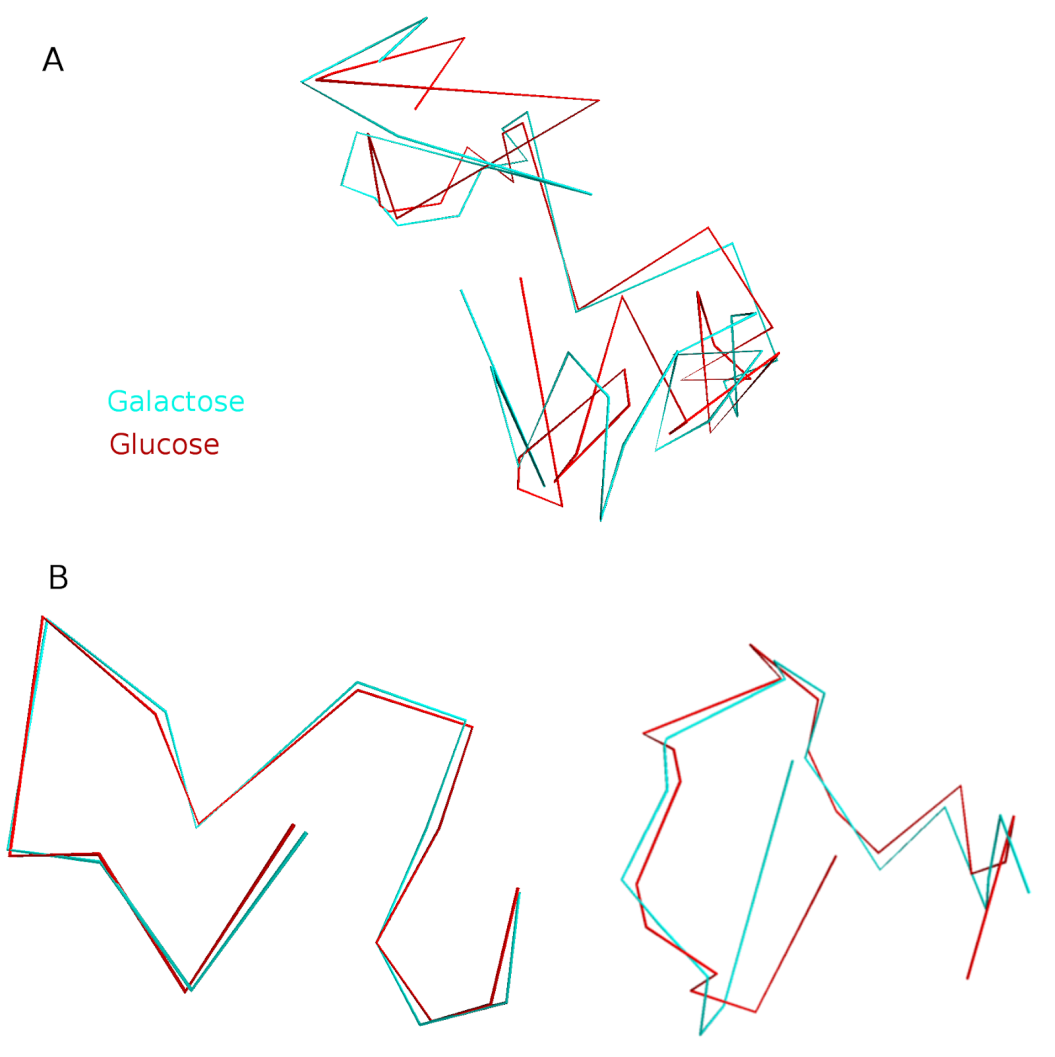


**Supplementary Figure 10.** chr12 *S. cerevisiae* glucose (red) and galactose (cyan) alignment for **A)** full structures and **B)** split structures.


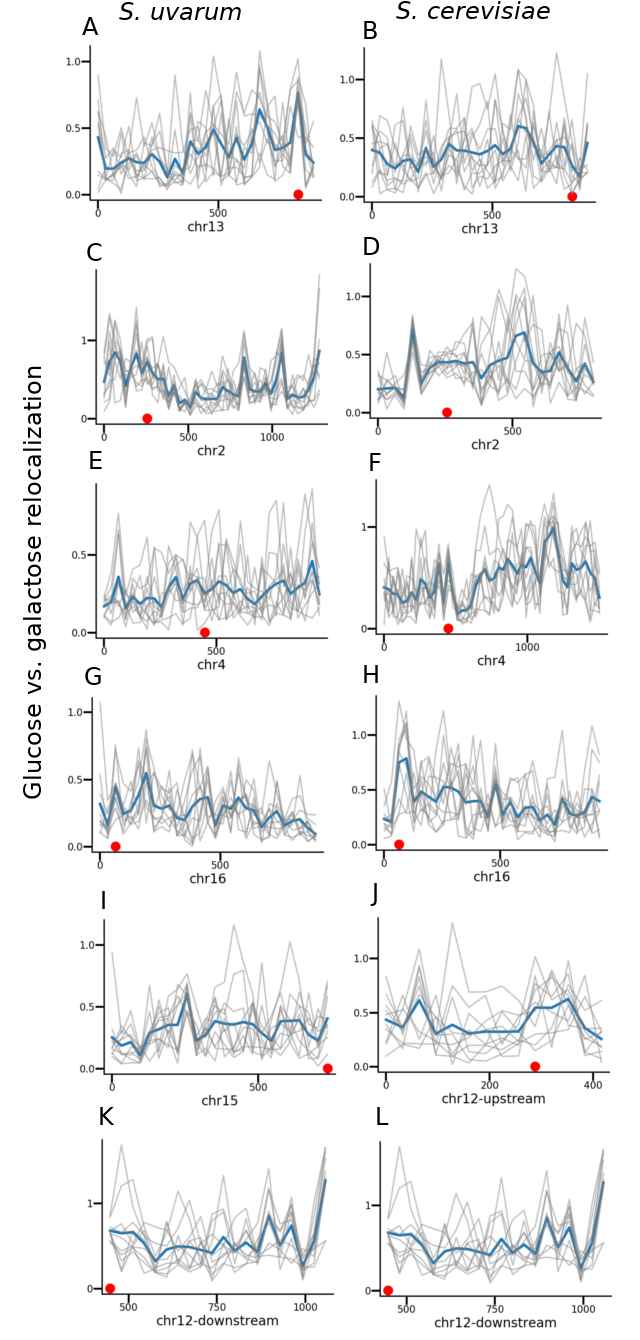


**Supplementary Figure 11.** Relocalization of yeast loci between glucose and galactose conditions calculated from independent MDS inference and alignment, separated by homolog, for selected chromosomes, calculated from individual inference and alignment. Gray: individual iterations. Blue: mean. Genomic coordinates (kb) are shown on x axes. **A-B:** Has1-Tda1. **C-D:** Gal1-Gal7-Gal10. **E-F:** Gal3. **G-H**: Gal4. **I-J:** Gal2. **K-L**: rDNA.


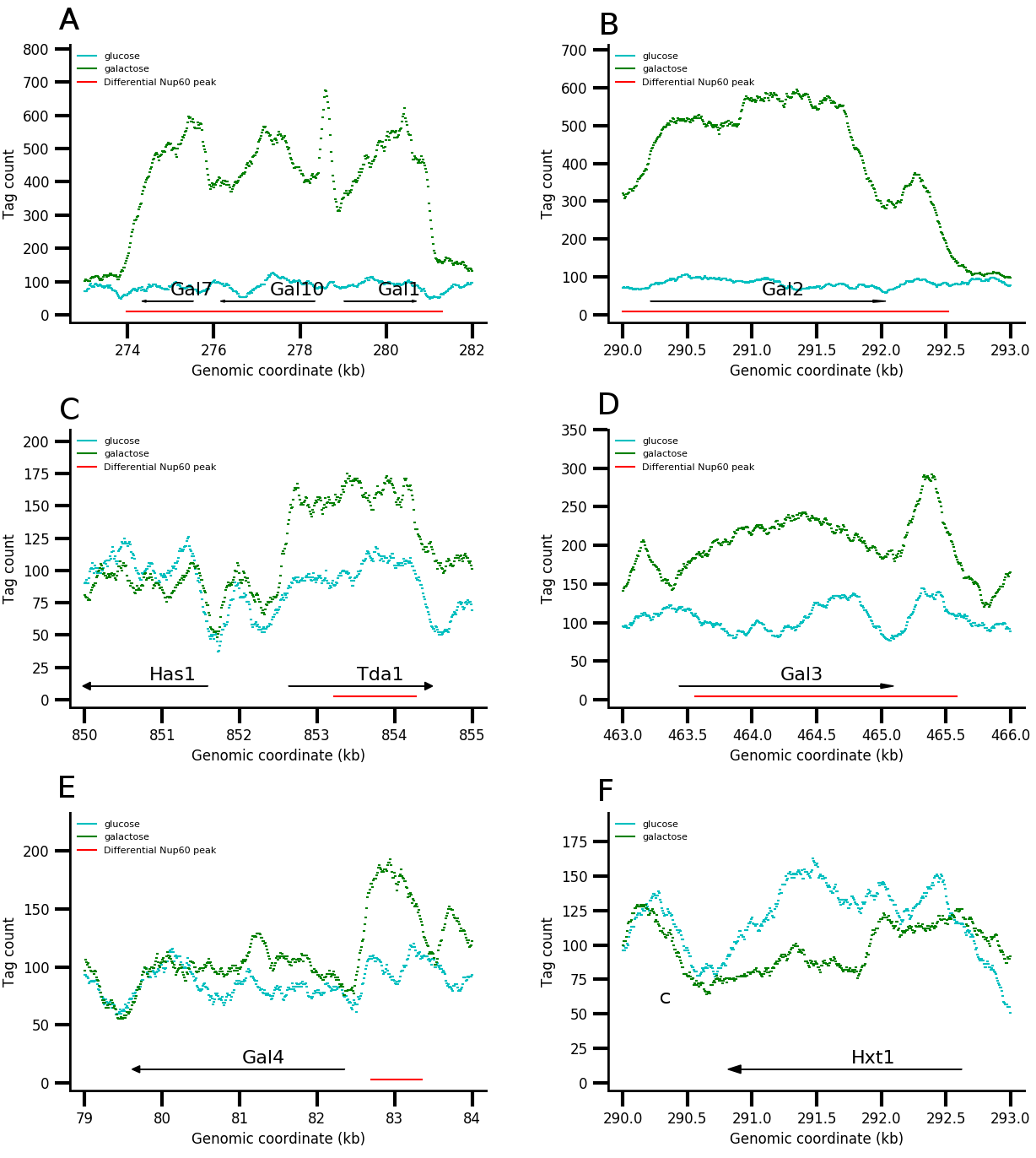


**Supplementary Figure 12.** Nup60 ChIP-seq tag counts in glucose and galactose conditions at **A)** Gal1-Gal7-Gal10, **B)** Gal2, **C)** Has1-Tda1, **D)** Gal3, **E)** Gal4, and **F)** Hxt1.


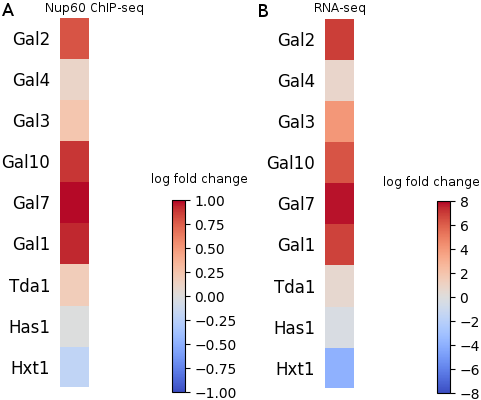


**Supplementary Figure 13.** Log fold change of **A)** Nup60 ChIP-seq tag counts and **B)** RNA-seq counts at selected genes in galactose relative to glucose.


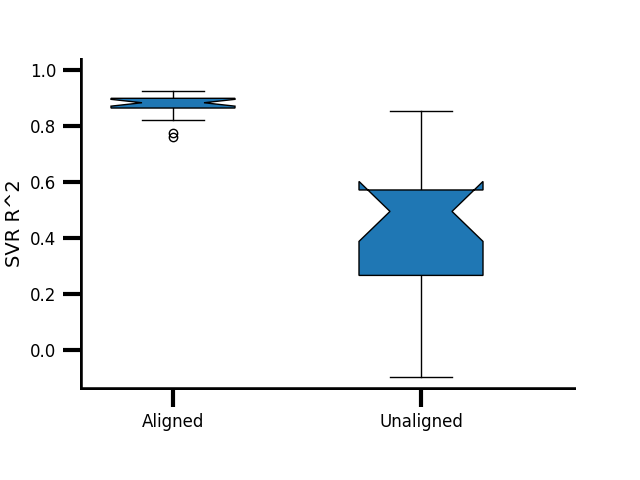


**Supplementary Figure 14.** SVR coefficient of determination of compartment scores predicted by 3D coordinates for aligned and unaligned GM12878 and K562 structures for each chromosome.


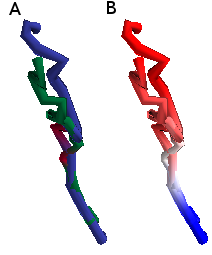


**Supplementary Figure 15.** chr21:41900000-43900000 aligned to the compartment axis (see Supplementary Fig. 7). **A)** Blue: GM12878. Green: K562: Red: Mx1/Mx2. **B)** Compartment scores. Positive scores are plotted in reds, and negative scores are plotted in blues.


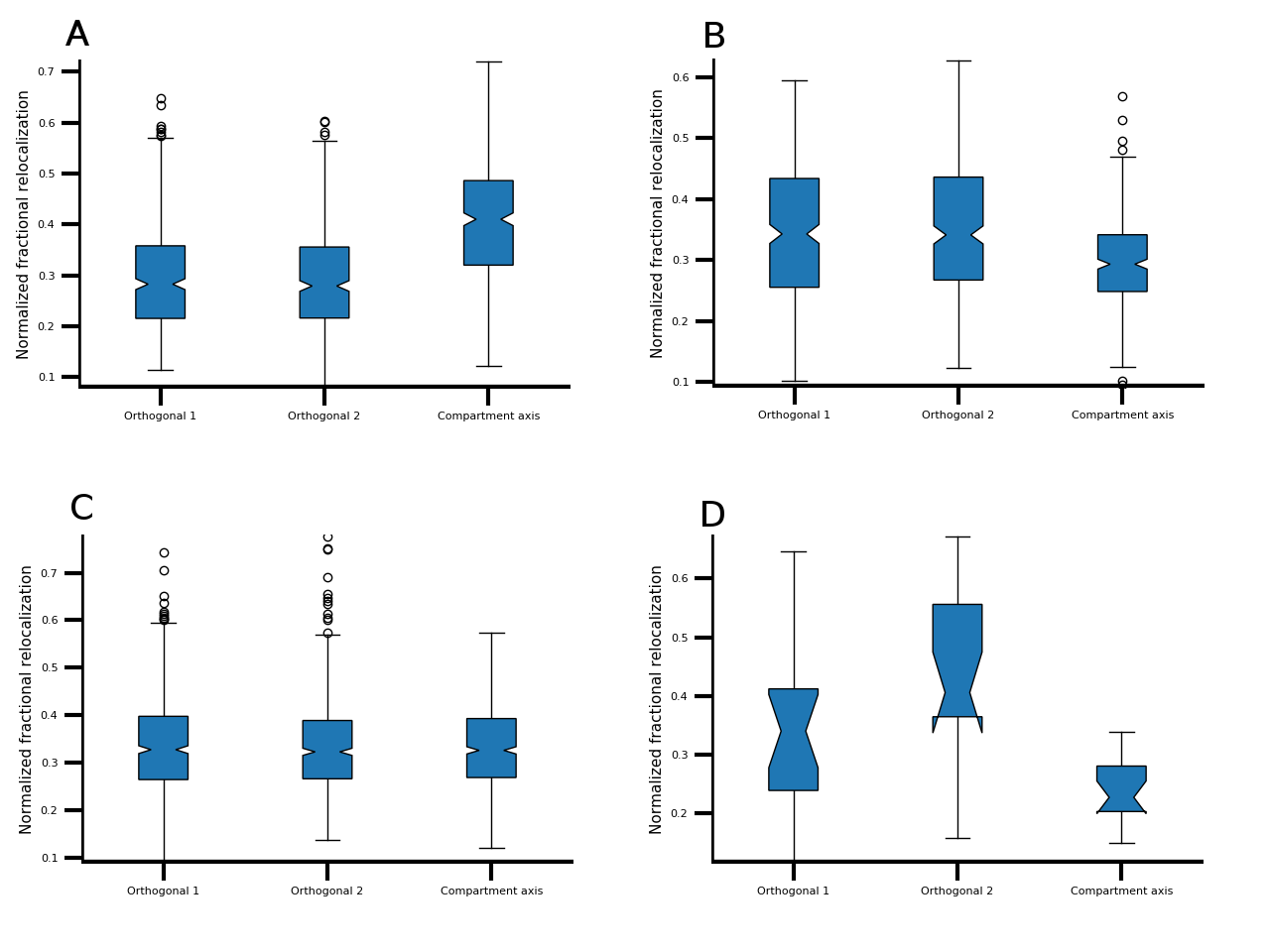


**Supplementary Figure 16.** Fraction of total relocalization distance along each axis for all chromosomes, calculated from independent structural inference and alignment, for **A)** ENCODE cell lines, **B)** mouse cell types, **C)** LCLs, and **D)** cohesin KO.


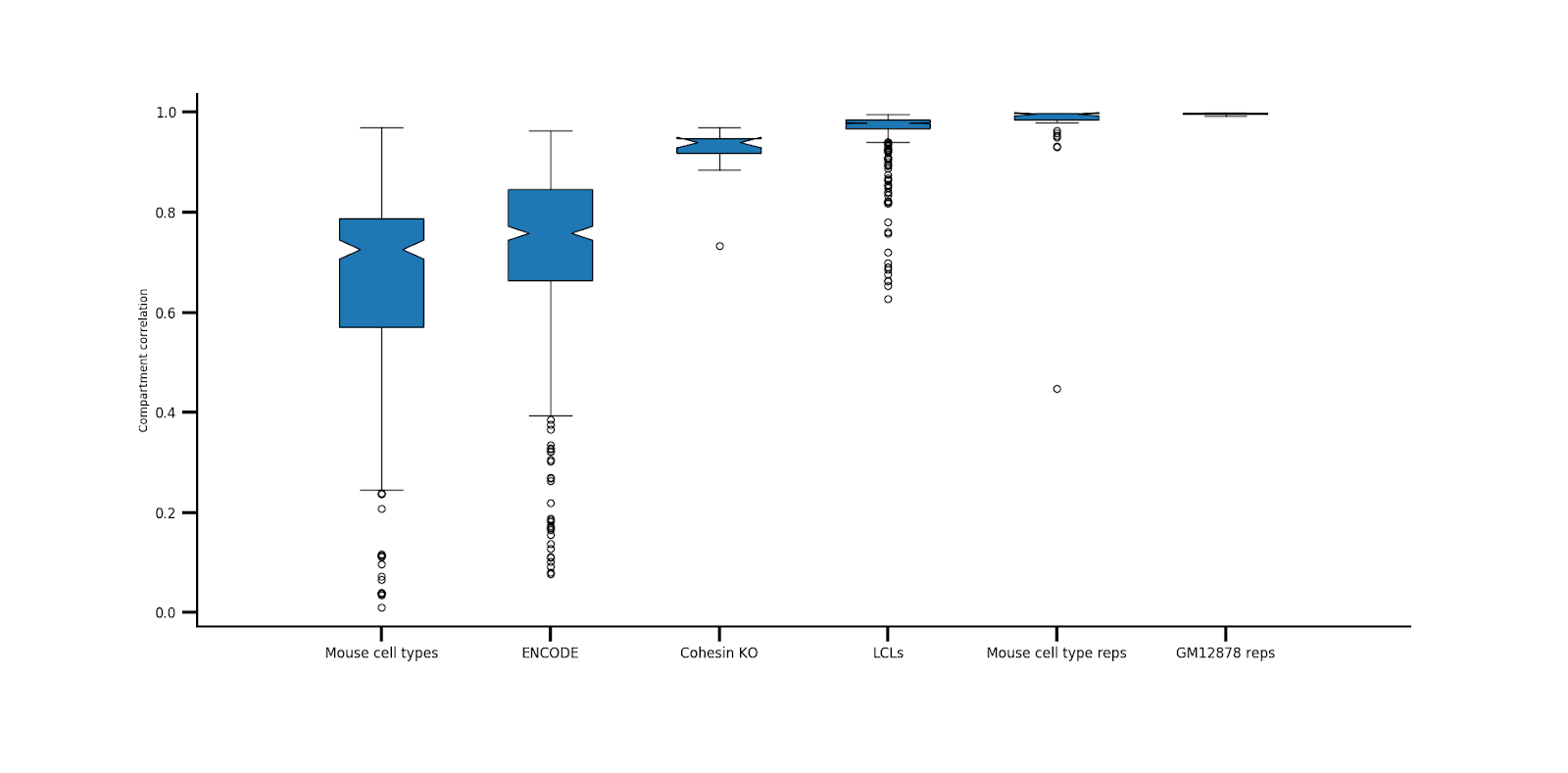


**Supplementary Figure 17.** Compartment score correlations between each pair of datasets, separated by data type.


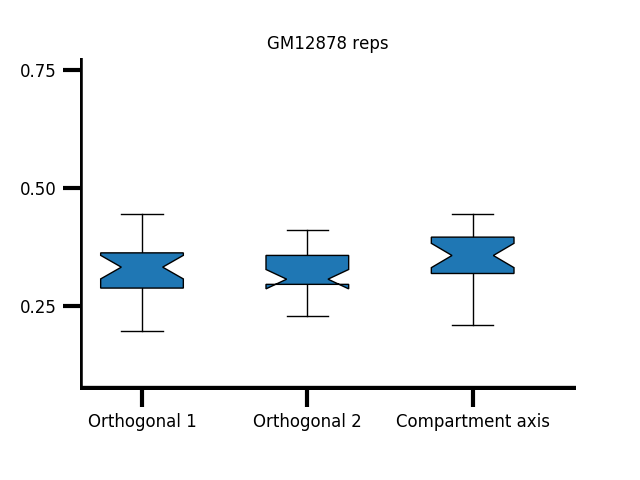


**Supplementary Figure 18.** Fraction of total relocalization distance along each axis for all chromosomes for GM12878 replicates.


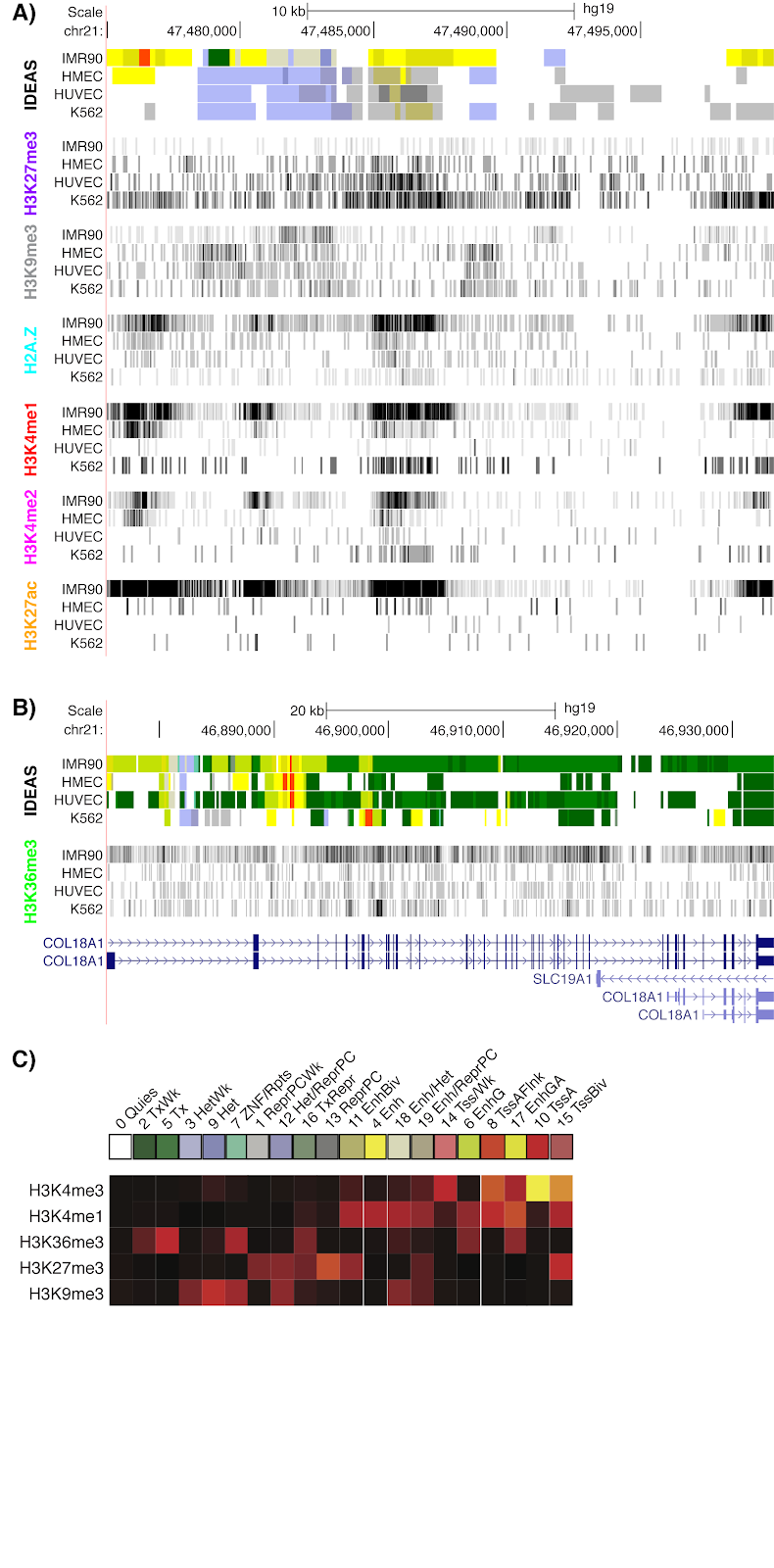


**Supplementary Figure 19. A)** ChIP-seq data for chr21:47.475-47.5 Mb. **B)** H3K36me3 ChIP-seq data for COL18A1. **C)** Emission probabilities for IDEAS states.


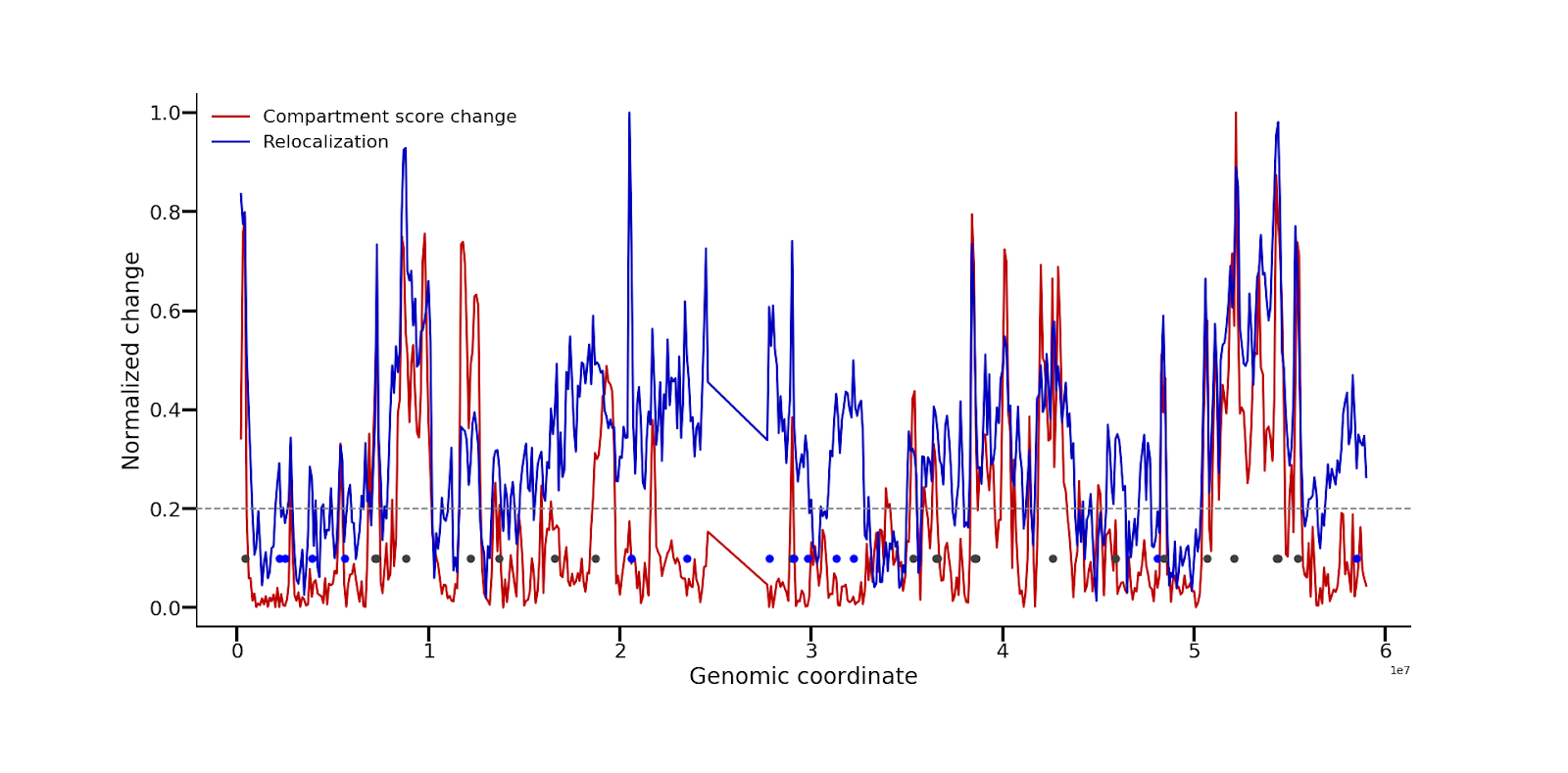


**Supplementary Figure 20.** Relocalization and absolute compartment score differences between GM12878 and K562 chr19, normalized to 1. For ease of visualization, 100-kb resolution is shown. Gray dashed line is the threshold for compartment-independent relocalizations. Gray dots are compartment-dependent relocalizations, and blue dots are compartment-independent relocalizations.


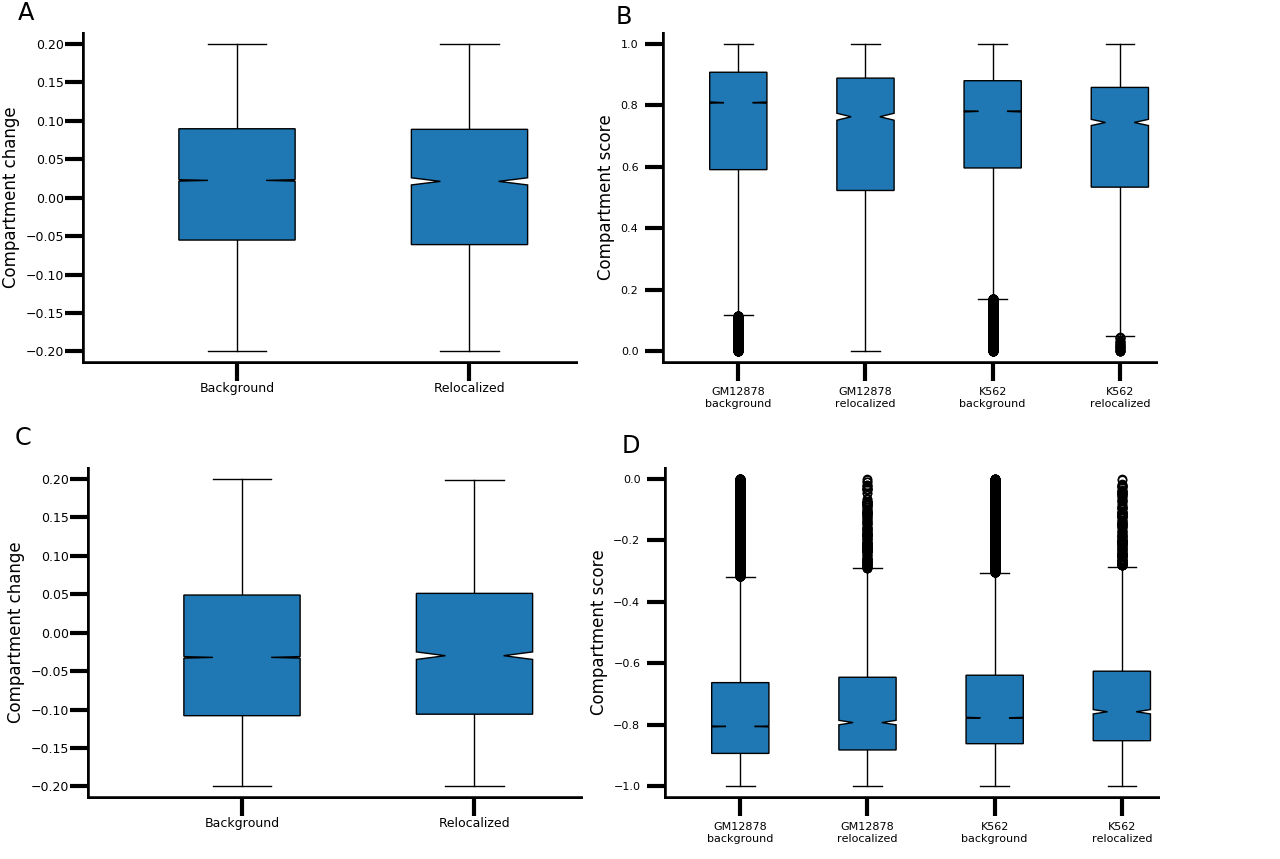


**Supplementary Figure 21.** Validation that compartment-independent relocalizations are not different from background in compartmentalization. **A)** Difference in compartment score in 10-kb A compartment bins that did not significantly relocalize and compartment-independent relocalizations. **B)** Compartment scores for both cell types in 10-kb A compartment bins that did not significantly relocalize and compartment-independent relocalizations. **C)** Difference in compartment score in 10-kb B compartment bins that did not significantly relocalize and compartment-independent relocalizations. **D)** Compartment scores for both cell types in 10-kb B compartment bins that did not significantly relocalize and compartment-independent relocalizations.


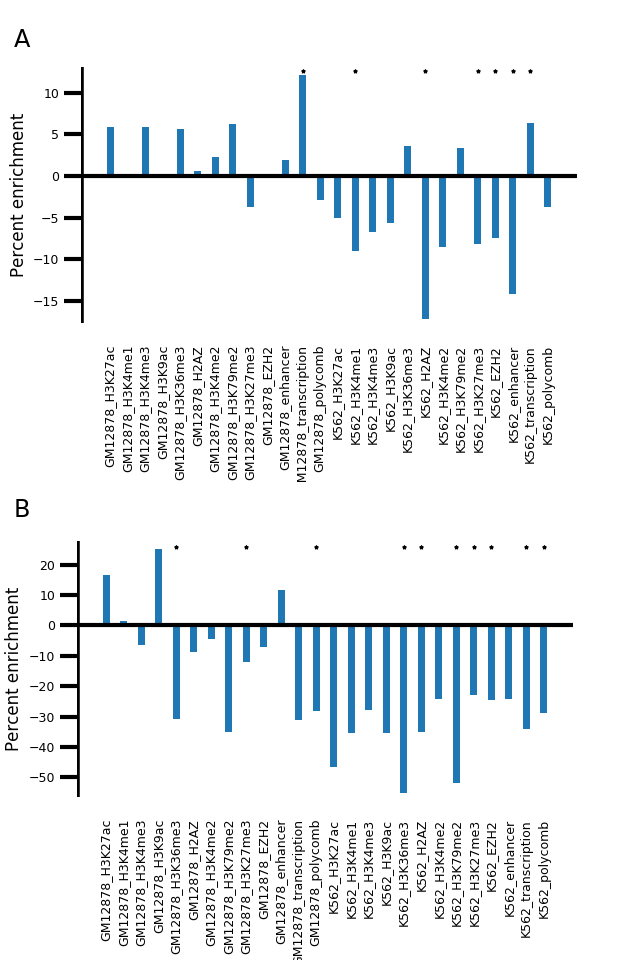


**Supplementary Figure 22. A)** Enrichment of mean coverage of selected chromatin marks in A compartment loci that relocalize without changing compartment score relative to those that do not relocalize, calculated from independent structural inference and alignment. **B)** Enrichment of mean coverage of selected chromatin marks in B compartment loci that relocalize without changing compartment score relative to those that do not relocalize, calculated from independent structural inference and alignment.


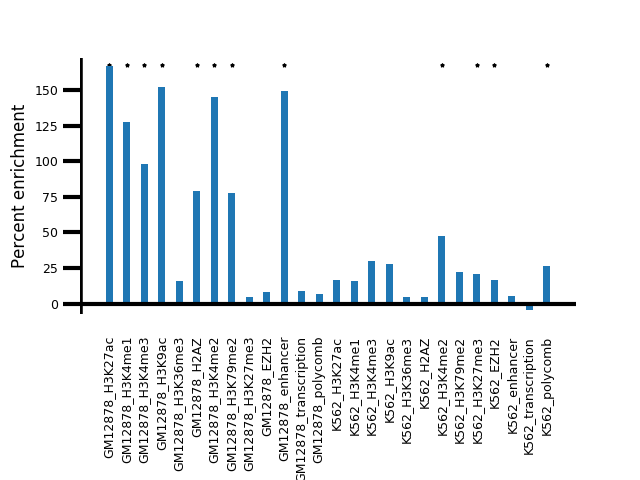


**Supplementary Figure 23.** Enrichment of mean coverage of chromatin marks in intra-B relocalization peaks relative to background B compartment. Stars represent *p*<0.01.


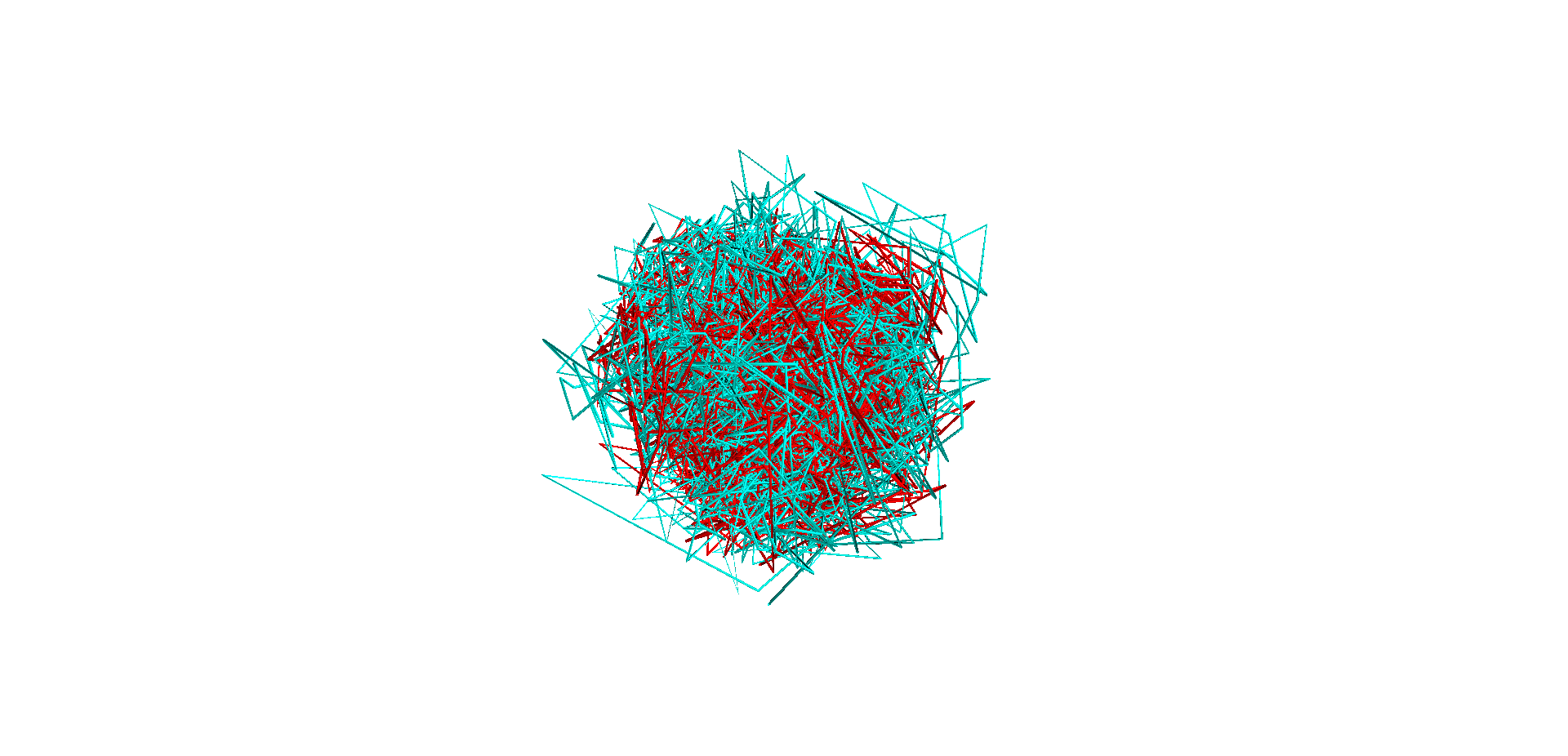


**Supplementary Figure 24.** MultiMDS applied to GM12878 (red) and K562 (cyan) chr21 datasets at 10-kb resolution without distance-decay prior. Compare to Fig. 1A, which is the output of the same analysis with a distance-decay prior of 0.05.

**SUPPLEMENTARY TABLE**

**Supplementary Table 1.** Compartment scores at chr21:47.4-47.5 Mb

| **Cell type** | **Compartment score** |
| --- | --- |
| IMR90 | 0.900 |
| HMEC | 0.756 |
| HUVEC | 0.767 |
| K562 | 0.853 |
